# Towards Cost-Effective and Sustainable Media Formulations for Terrestrial and Aquatic Cellular Agriculture

**DOI:** 10.3390/foods15142494

**Published:** 2026-07-14

**Authors:** Regina Leber, Joana T. Rosa, Vincent Laizé, Gonçalo F. Fernando, Johannes Buyel, Aleksandra Fuchs

**Affiliations:** 1 ACIB—Austrian Centre of Industrial Biotechnology, Petersgasse 14, 8010 Graz, Austria; 2 S2AQUA—Collaborative Laboratory, Association for a Smart and Sustainable Aquaculture, Av. Parque Natural da Ria Formosa s/n, 8700-194 Olhão, Portugal; joana.rosa@s2aquacolab.pt; 3 CCMAR—Algarve Centre of Marine Sciences, University of Algarve, Campus de Gambelas, 8005-139 Faro, Portugal; vlaize@ualg.pt; 4 Associate Laboratory i4HB—Institute for Health and Bioeconomy, Instituto Superior Técnico, Universidade de Lisboa, Av. Rovisco Pais, 1049-001 Lisbon, Portugal; goncalo.fernando@tecnico.ulisboa.pt; 5 Institute of Bioprocess Science and Engineering (IBSE), Department of Biotechnology and Food Sciences (DBL), BOKU University, Muthgasse 18, 1190 Vienna, Austria; johannes.buyel@boku.ac.at; 6 Division of Physiology, Otto Loewi Research Center for Vascular Biology, Immunology and Inflammation (E.O.), Medical University of Graz, 8010 Graz, Austria

**Keywords:** cultured meat, cultured seafood, cellular agriculture, serum free media formulations, cost reduction strategies, key performance indicators

## Abstract

Over a decade of research on media for cultured meat and seafood production has resulted in multiple highly efficient serum-free and chemically defined formulations for some species, but it has also identified challenges yet to be solved—especially for aquatic cell lines. Depending on the product and cell type, the approach to develop highly efficient, sustainable, and low-priced media can diverge greatly. In this review, we provide an in-depth overview of this complex research area to facilitate strategic decision-making for stakeholders. We evaluate the advantages and limitations of utilizing hydrolysates, growth factor mutants, growth factor alternatives, and stabilizers in serum-free media formulations published for cultured meat production, as well as ongoing research efforts on developing adequate media for cultured seafood. We critically analyze strategies aimed at reducing medium costs and enhancing sustainability of cultured meat and seafood production, including their food-compatibility assessment. We summarize topics that require further exploration, such as identification of species-specific growth factors—particularly for aquatic species; exploration of hydrolysates as a substitute for basal medium; waste medium recycling strategies; and the potential application of artificial intelligence (AI) and machine learning (ML) technologies to enhance these areas. Additionally, we consider possible emerging regulatory issues and their impact on media formulation development. Finally, key performance indicators for media formulations are proposed to guide future strategic and operational improvements regarding an economical and sustainable production process.

## 1. Introduction

Meat and seafood are playing crucial roles in human nutrition because they are rich sources of high-quality protein [1]. Global demand for animal-derived protein has already increased substantially over the past decades and is projected to continue rising [2]. Although there are plant-based “meat” analogue products available, genuine meat will remain the preferred protein source for most people in the near future [3]. Herd sizes are projected to go up by a further 20% until 2050 to meet the growing global demand for animal proteins [4]. Furthermore, global seafood consumption has doubled since 1960 and it will continue to increase [5], but the conventional way to produce meat and seafood cannot meet this high demand without having significant environmental impacts [6].

Cultured meat (CM) and cultured seafood (CSF) are produced by cultivating animal cells *in vitro* [7]. CM is increasingly integrating into the mainstream food industry as a new method of producing animal protein for human consumption. It has already entered the Singapore market, with GOOD Meat cultured chicken available since late 2020, and Vow’s quail product available since 2024, along with several other companies in development (discussed in detail in Section 5).

Efficiency of cellular agriculture—which is expressed by how much input mass (mainly medium components) is converted into desirable product mass—is largely determined by the culture conditions, cell type and its respective cell doubling time, cell culture medium and its biomolecular composition, and the type of bioreactor used [3]. This review focuses on one of these factors—serum-free media (see Box 1)—as it is an absolutely pivotal and highly cost-intensive factor in the production of CM and CSF, accounting for 55–95% of production costs [6,8]. This review consolidates recent research, highlights ongoing challenges, unveils new bottlenecks, and suggests solutions thereof to facilitate the orientation of the stakeholders and accelerate progress to a highly cost-efficient, safe, food-compatible, scalable, and sustainable media for CM and CSF. The primary focus of this review is on vertebrate livestock (e.g., bovine, porcine, avian, etc.) and aquatic species used for CM/CSF—the latter were to our knowledge never reviewed before.

Box 1 **Definitions.**

⇨ **Serum-free**—formulations that do not contain animal or human serum but may include purified or recombinantly produced components, such as serum albumins.
⇨ **Animal component-free**—formulations that do not contain components derived from animals directly but may include their recombinantly produced equivalents.
⇨ **Chemically defined**—a composition where all chemical components are known, precisely identified, and present in exact, specified amounts.
⇨ **Food-grade**—components meet regulatory safety standards set by food authorities (e.g., EFSA in the EU, FDA in the USA), and are thus approved as safe and suitable for use in food intended for human consumption.

Although meat is mainly composed of muscle tissue, fat typically accounts for up to 10% of biomass [9]. Fat is the most important contributor to meat flavor across all species [10,11,12], and will most likely be part of the first CM hybrid products reaching the market in Europe [13]. Thus, **additionally to muscle**, we included fat as one of the designated final products, and reviewed serum-free media formulations for **terrestrial and aquatic adipocyte precursors**.

Generally, three main categories of cell types with diverging media preferences can be considered for CM/CSF production, and are reviewed in this article: (1) adult progenitors (e.g., here mentioned primary cells, muscle satellite cells, fibro-adipogenic progenitor cells (FAPs) and mesenchymal stem cells (MSCs)), which can produce limited range of cell types; (2) pluripotent stem cells (PSCs, including induced pluripotent stem cells (iPSCs) and embryonic stem cells (ESCs)); and (3) immortalized cell lines. Cells isolated from various species, as well as from diverging sex, breed, and age of the animal may show differences in medium requirements [14,15,16]. Finally, continuously proliferating cell lines can be adapted to tolerate less rich/expensive medium formulations [17]. Thus, we focus on the media compositions for the aforementioned cell types throughout the entire production process—from **isolation and cryopreservation to differentiation and maturation**—which has not been previously documented as a comprehensive overview.

We further review possible strategies that may also contribute to a cost reduction and higher sustainability for cell culture media—including food-compatible growth factor excipients used in a culture medium, which were not reviewed previously—and discuss regulatory issues and key challenges that remain to be addressed.

An emerging area of interest, involving insect cell culture for food applications, falls outside the main scope of this review, and is reviewed elsewhere [18,19]. Additionally, only fully declared media compositions were reviewed, as it is a prerequisite for further medium optimisation efforts.

## 2. History of Serum-Free Cell Culture Media Development for CM

Traditionally, cell culture media are supplemented with animal serum (particularly fetal bovine serum, **FBS**) to support animal cells *in vitro*. FBS contains essential components required for proliferation and maintenance of mammalian, as well as fish cells. Most important are proliferation-promoting proteins like hormones and growth factors (GFs) [20]. Although FBS is traditionally a universal supplement for cell culture media, it has several **limitations** and there are rising **ethical concerns** as FBS is obtained from the foetuses of pregnant slaughtered cows. FBS is a byproduct of the meat industry, and its **availability** may be subject to fluctuations caused by climate change, contagious diseases, limited water supply or extreme weather events [21]. Additionally, as the pharmaceutical industry is currently the largest user, the growing CM/CSF industry would have to compete with it, potentially resulting in **even higher prices and increased availability challenges** due to the rising demand.

As a biological component, FBS can be a source of contamination by viruses or other infectious agents, which can raise **concerns over the safety** of FBS for food production. In Europe, FBS must be tested for seven specific viruses according to the Code of Federal Regulations Title 9 (9CFR) guidelines to avoid potential pathogen transmission [22]. However, researchers have detected viruses in commercial FBS batches [23,24], which may raise **regulatory concerns**.

Further, the exact **composition of FBS is unknown** and has batch-to-batch differences depending on its origin and processing, which may impact on the reproducibility of cell culture processes [25].

Despite these limitations and its high cost, FBS is often a major constituent of cell culture media (typically 2–20% by volume). Hence, the development of **SF medium formulations** has gained much attention over the last decades (see the detailed review by Yao and Asayama on the history of the transition from FBS-containing to SF media [26]).

The first developments in this regard were achieved by Chen et al. when identifying seven components including two GFs to be essential for human ESC and iPSC cultures [27]. These seven compounds were added to the chemically defined basal medium DMEM/F12. The resulting so-called **Essential 8 medium** (E8) is an SF cell culture medium providing chemically defined conditions for human cells used in research and clinical applications [27]. The E8 medium was further optimized (and named B8 medium) regarding medium constituents and concentrations required to culture human PSCs by Kuo et al. [28]. Costs of the **B8 medium** could be substantially reduced by the in-lab generation of three *Escherichia coli*-expressed, codon-optimized recombinant GFs (fibroblast growth factor 2—FGF-2, transforming growth factor β3 (TGFβ-3), and neuregulin 1(NRG-1)) [28].

These formulations optimized for human cell lines provided the starting point for developing SF-media for CM production, with most of them optimized for muscle satellite cells (SCs) [29,30,31]. For other cell types such as ESC and iPSC [32], less medium optimization efforts in the serum-free medium were reported so far, except for some information for porcine ESCs and iPSCs [33,34].

For aquatic species, research in this area is still in its early stages, with initiatives focusing on **low serum and SF proliferation media for muscle-derived cell lines and fibroblasts** often yielding suboptimal results. The research is further complicated by the fact that bovine serum is not an ideal proliferation promoting medium component for fish cells. When FBS, routinely used in fish cell culture media, was replaced with gilthead seabream (*Sparus aurata*) serum, **seabream mesenchymal-derived cells** VSa13 and VSa16 performed significantly better regarding proliferation and extracellular matrix mineralization [35]. Additionally, **several embryonic stem cells** require fish embryo extracts [36,37] or fish serum [38] for their isolation and proliferation in addition to FBS. Both cellular and serum components may be sourced from the same or different fish species [39,40,41]. Similarly, **goldfish muscle cells** [42] rely on **species-specific muscle extracts and fish serum**, with fish serum demonstrated to be essential for their long-term viability [43].

While human and bovine GF orthologs share 90 to 98% protein sequence identity, allowing an efficient culture of human cells in bovine serum, the protein sequence identity between fish and bovine GF orthologs, as well as between fish orthologs, is in the range of 77% [44], which may result in the suboptimal proliferation of many types of fish cells in FBS or in fish serum from distant species. This highlights the importance of using species-specific serum or extracts, rather than FBS, to efficiently support the proliferation of fish cells. It should be noted that fish serum/extracts also exhibit some of the limitations associated with FBS, such as limited availability, batch-to-batch variability and ethical acceptability, which impede their application in the production of CSF. Collectively, these findings underscore current **critical reliance of fish cell lines on FBS and fish extracts**. **More research** is needed to identify **species-specific GFs,** critical for different developmental stages of fish cells, before their dependence on FBS and fish extracts can be eliminated.

A recent landmark study by Jozef et al. demonstrated, for the first time, that a systematically developed, fully defined and transparent SF medium can sustain long-term proliferation of a fish cell line, the rainbow trout gill cell line RTgill-W1, for at least 35 passages, including successful cryopreservation [45]. While neither the cell type nor the intended application is directly relevant to CSF production, this work provides an important methodological framework and proof-of-concept that fully defined SF media for fish cells are achievable.

Given the high demand for different cell types and species (especially for fish), a universal chemically defined SF medium for meat or fish cell cultures appears to be unrealistic [46,47]. Thus, the development of low-cost, customizable and sustainable media allowing optimal proliferation and differentiation of cells for the large-scale production of high-quality CM/CSF products remains a big challenge.

A **possible alternative** for medium optimization for every cell line is the **spontaneous adaptation** of cells to SF and generally less nutrient-rich media conditions. This approach has been successfully employed in the context of fish cell lines not directly relevant to CSF [48,49,50,51,52] but also to reduce FBS content from 20% to 2.5% in the medium for the culture of mackerel muscle cell line Mack1 [53]. This approach was also successfully used to **spontaneously immortalize chicken fibroblasts** capable of adipogenesis [54]. While proliferation of bovine SCs in SF medium requires multiple GF supplementation, chicken fibroblasts derived from several chicken breeds were adapted to not only grow in single cell suspension, but also in an albumin-free and SF culture medium containing practically no GFs (see Supplementary Table S1 for contents and cost comparison).

These studies report **methodologies** that may be adapted to the development of SF media for other meat- and seafood-relevant cells. While attractive from a media development point of view, this approach, by relying not only on metabolic changes but also on random mutagenesis, harbors **serious limitations**—such as ineffectiveness, collection of large number of background mutations, and very limited screening possibilities for the required phenotypes [55]. For instance, attempts to adapt the **silver seabream myofibroblast precursor** Catmus1PFR cell line to lower FBS concentrations resulted in a progressive decline in cell proliferation, ultimately leading to cell death upon complete removal of FBS [56]. Similar trends were observed in other fish cell lines [57]. In addition, the adaptation to SF conditions of cell lines from bigger animals may not work out that well as **spontaneous immortalization of mammalian cells has been negatively correlated with species body mass** [58], which makes this method more applicable to small animals.

## 3. Media Compositions from Cell Isolation to Maturation

The process of CM/CSF production can be divided into four phases, namely: (i) cell isolation and maintenance; (ii) generation of cell biomass (proliferation); (iii) cell differentiation and maturation into skeletal muscle cells and fibres, or adipocytes and (iv) assembly of the final meat or fish product.

The successful cell culture of animal cells in an *ex vivo* environment under controlled conditions requires an appropriate medium providing a source of energy and essential nutrients like amino acids, vitamins, and inorganic salts [59], whereas a buffer system is necessary to maintain a physiological pH over time [60]. For most cell types, additional supplementation with cell type- and phase-specific proliferation promoting factors like GFs and/or hormones is crucial for SF media to be effective [47,61].

Thus, the **choice of cell type and final product will greatly influence the media requirements**, and, consequently, the **price** of the media, as evidently shown in Supplementary Tables S1–S3. For an economically viable CM and CSF production process it is important that the final formulations are cost-efficient, food-safe, and scalable [29].

Further, contamination by microbes can be hazardous to cell culture systems used in the CM/CSF sector as contaminated products must be discarded. Supplementing proliferation media with antimicrobial agents such as antibiotics and antifungals generally provides an effective method to prevent contamination. However, antibiotics are biologically active and exhibit concentration-dependent effects on mammalian cells [62]. Therefore, prophylactic antibiotics supplements should be limited to cell isolation processes. During proliferation and differentiation, microbial contamination can be prevented by following standard aseptic techniques which have been established in pharmaceutical industry decades ago. Following this, we do not address the impact of antimicrobial agents on cell proliferation and differentiation, nor do we include their costs in media cost comparisons.

### 3.1. Media for Cell Isolation and Storage

During cell isolation, a medium composed of basal medium supplemented with 1% antibiotics and antimycotics to prevent contamination, and **10**–**20% FBS** (see Table 1 and Box 2) is usually used. To our knowledge, no SF isolation medium has been published so far. After the initial mincing, the tissues are enzymatically digested to produce single cell suspension (see the overview of the enzymes used for isolating CM-relevant satellite cells in [63]), which is eventually stopped by the addition of FBS. Aside from factors necessary for cell maintenance and proliferation, FBS also contains protease inhibitors like α1-antitrypsin and α2-macroglobulin [64], which are important for ending protease treatment during cell isolation. Studies evaluating the possibility to replace FBS by protease inhibitors for cell isolation processes are lacking, which might be one of the reasons why SF isolation media were not developed so far.

Box 2 **No published SF media for primary CM/CSF-relevant cell isolation and storage under food-grade constraints.**

⇨ **No SF isolation medium** has been published so far.
⇨ **Chemically defined** and **SF cryopreservation media** are commercially available, but composition is not publicly available.
⇨ **Food grade** chemically defined and SF cryopreservation media are required on the market.

For long-term storage, isolated animal cells are routinely **cryopreserved** at ultra-low temperatures (<−150 °C) in the presence of a range of cryoprotectants. In reports mentioned in Table 1, mammalian cells were suspended in basal medium containing **10% FBS** and 10% (vol/vol) dimethyl sulfoxide (DMSO) [66,69,71] or frozen in FBS with 10% DMSO [31,65,73]. Chemically defined and **SF cryopreservation media** with and without DMSO, which provide a safe and protective environment during the freezing, storage, and thawing process for mammalian cells, are available on the market. Their composition, however, is proprietary and it is unclear if the ingredients can be rated as food-grade. Interestingly, plant hydrolysates have been successfully used to cryopreserve CHO cells, which improved cell survival during the freezing and thawing [77]. Additionally, significant effort was dedicated to developing serum-free or DMSO-free cryopreservation media for various CM-irrelevant cell lines, which should be adaptable for use in the CM field. In this regard, similar to stabilization of proliferation media (Section 4.4.5), food-relevant polysaccharides (e.g., dextran, methylcellulose, alginate) and other polymers (e.g., hyaluronic acid) were successfully tested as substitutes of FBS and/or DMSO [78,79,80].

The cryopreservation of **fish cells** relies on methods and reagents originally developed for mammalian cells. A standard formulation of the cryopreservation medium contains 10% DMSO and 10–50% FBS, with 20% FBS emerging as an optimal concentration to boost cell recovery [81,82,83]. For cells requiring an enhanced protection, DMSO concentration may be increased to 20% in combination with 20–40% FBS [84,85]. Conversely, less sensitive cells may be cryopreserved in 5% DMSO and 25% FBS [86]. Glycerol has been reported in the past as an effective cryoprotectant (as an alternative or a complement) and is typically utilized at concentrations ranging 5–10% [87]. These different protocols highlight the importance of tailoring cryopreservation to the unique needs of each fish cell line, ensuring optimal post-thaw viability and functionality.

### 3.2. Media for Cell Proliferation

Several serum-free media formulations have been published for growing cells derived from **terrestrial animals** *in vitro* and have been very shortly reviewed by Quek et al., 2024 [88]. These media are based on DMEM/F12 basal media, which was originally identified to be the best choice for human cells by Chen et al. [27]. DMEM/F12 is a 1:1 mixture of DMEM and Ham’s F12 medium and uses sodium bicarbonate as buffering system to maintain a physiological pH. Alternatively, HEPES is included to support the buffering capacity of DMEM/F12 [89]. This basal medium typically requires supplements to support and maintain cell proliferation. The components as well as their concentrations vary depending on the cell type and species (see overview in Supplementary Table S1 and Box 3).

Box 3 **No universal chemically defined proliferation medium under food-grade constraints is published so far, applicable to all CM/CSF cell types and species.**

⇨ SF proliferation media for **muscle stem cells and fibro-adipogenic progenitor cells from terrestrial species** have been developed.
⇨ SF proliferation media for **CM/CSF unrelated ESCs/(i)PSCs** are available, but lack for species considered for consumption.
⇨ Proliferation of **aquatic muscle and fat precursors still depends on media supplemented with serum**.
⇨ Cell line adaptation leads to extremely cost-effective media solutions, but risk factors have to be considered.

Leibovitz’s L-15 is used as basal medium for the cultivation of a wide range of **fish cells**, but recent studies revealed that F10 and DMEM/F12 could be more suitable for fish primary SCs, with F10 demonstrating a more pronounced effect on proliferation compared to DMEM/F12 [90]. Notably, **marine fish cells** typically require a **higher osmolarity** compared to both freshwater fish and mammals [91,92,93].

It is clear from world-wide efforts that there is no single alternate or chemically defined medium or even a formulation of a group of components that can replace FBS in culture medium for all possible cell types [20]. Still, several published compositions proved to be efficient for multiple CM-relevant cell lines and can be taken as a basis for specific cell line adaptations.

In the following sections, we summarize the efficacy of SF-media for different cell types.

#### 3.2.1. (Induced) Pluripotent Stem Cells (iPSCs) and Embryonic Stem Cells (ESCs) of Terrestrial Species

Human PSC systems, briefly reviewed here, provide a mature framework for chemically defined, SF culture, from which CM/CSF media development can draw. As shortly mentioned in Section 2, **Essential 8 (E8)** is a widely used and commercially available animal component-free medium, originally designed for **human** PSC and ESC culture [27]. It works well for expansion and prolonged maintenance of stem cells without triggering differentiation [27,94]. E8 is based on the basal medium DMEM/F12 supplemented with L-ascorbic acid-2-phosphate, magnesium, sodium selenium, FGF-2, insulin, sodium bicarbonate, transferrin, transforming growth factor (TGF)β1 or Nodal [27,95] (for their concentrations and costs see Supplementary Table S1).

The concentrations of the eight components in E8 were further optimized to support a higher proliferation rate of human iPSCs under low seeding density conditions [28]. The resulting medium formula termed B8 could support both hiPSC generation and long-term culture for more than 100 passages (see composition in Supplementary Table S1). Observations of the authors and communications with other researchers led to the conclusion that neither E8 nor B8 media were universally suited for all human iPSC lines.

Multiple commercial SF medium compositions were shown to support the proliferation of iPSC/ESCs from species considered for consumption [96,97], but their full list of components is not revealed, which naturally hampers further media optimization efforts. Such commercial SF media are quite expensive, and the possibility of their upscaling is questionable. Another example is the medium published for porcine iPSCs, which includes three inhibitors known for enhancing human embryonic stem cells self-renewal (CHIR99021, SB431542, and PD0325901), and three cytokines (BMP4, SCF, and IL-6), but also contains human platelet lysates, which makes it not applicable for the CM/CSF area [33]. Significantly more data is available for human iPSCs, but such media compositions would require verification and possibly adaptation for iPSCs from species considered for consumption. For example, a simplified SF culture medium containing FGF-2 and the WNT and SRC pathway inhibitors IWR-1 and WH-4-023 [34] has been reported, but it is formulated using the commercial medium mTeSR™ with a proprietary composition, thus not ideal for CM/CSF applications. Thus, we state that there is a lack of chemically defined medium alternatives for proliferation of iPSCs and ESCs from species considered for consumption, with a fully declared media composition, which is a prerequisite for further medium optimisation efforts.

#### 3.2.2. Bovine Satellite Cells (BSCs) and Porcine Satellite Cells (PSCs)

Stout et al. adapted the SF medium B8 from Kuo et al. after they observed that BSCs isolated from a 2-week-old calf proliferated only for 3 days in this medium, indicating that a complete substitution of serum by B8 compounds did not meet the requirements of BSCs [28,31]. The addition of **recombinant human serum albumin** (rHSA, 800 mg/L) enabled BSCs to grow for at least seven passages over 28 days [31], underlining the importance of molecules with stabilizing properties, as also present in serum. This adapted B8 medium was termed **Beefy-9 or B9**, (see Supplementary Table S1) due to its component number and its specific design for bovine muscle cells. Increasing the concentration of albumin in B9 further improved the proliferation of BSCs in over one-month-long experiments of cell expansion. BSCs in B9 containing 3.2 and 6.4 mg/mL albumin reached 19 and 20 cell doublings within 28 days, respectively, as compared to 18 cell doublings for 0.8 mg/mL albumin [31]. However, higher albumin concentration also substantially increased costs for this medium. Further on, B8 stabilized with methylcellulose and B9, with and without methylcellulose, also demonstrated to perform well for both BSCs, PSCs, and chicken satellite cells (CSCs), with the more expensive B9 marginally underperforming in cases of PSCs and CSCs [98]. This line of research—stabilization of media—is further discussed below in the Section 4.4.5.

Kolkmann et al. performed a five-step medium optimization process, which led to reduced concentrations of all components present in B9 except for rHSA [29]. HSA was increased to 5 g/L and also the number of GFs was elevated (Supplementary Table S1). Consequently, costs for this medium are appr. 20× higher than for B9, with 20% of the total medium price attributed to HSA and appr. 60% to fibronectin (cell adhesion agent), and IL-6, HGF, and PDGF as the most costly GFs. This medium supported proliferation of BSCs over six passages similar to the control proliferation medium containing 20% FBS [29].

Skrivergaard et al. applied a Design of Experiment (DOE) approach in combination with a Response Surface Methodology to develop and optimize an SF medium for BSCs [30].

This approach reduced the number of constituents, especially of GFs. Instead of rHSA they used a combination of 75 mg/L serum-derived bovine serum albumin (BSA) and 600 mg/L recombinant fetuin. The fetal serum component fetuin is a serum carrier protein like albumin with multiple important biological functions including insulin signaling [99]. Fetuin had much higher proliferative effect on BSCs than BSA, but the authors were constrained to applying BSA at quite low concentration of 75 μg/mL, as higher amounts of BSA seemed to hinder cell attachment (also noted for HSA by Stout et al. [31]). The addition of 600 µg/mL fetuin and 75 µg/mL BSA to DMEM/F12 allowed a reduction of the amount of FGF-2 from 10 ng/mL [29] to 2 ng/mL [30]. The final minimal formulation of Skrivergaard et al. resulted in significant positive effects on the proliferation of BSCs over a 5-day period compared to the 10% FBS-containing control, and this medium also sustained proliferation of PSCs at a significantly higher level than 10% FBS in a short-term experiment. Costs for this medium are 2.5 times lower as compared to the medium from Kolkmann et al., but fetuin accounts for approximately 80% of the costs (see Supplementary Table S1) [30].

Similarly to Kolkmann et al., the inclusion of PDGF (5 ng/mL) and HGF (20 ng/mL) showed further positive effects in short-term proliferation assays with BSCs and PSCs, as well as in long-term cultivation of BSCs [30]. The same effect was reported by Schenzle et al. in methylcellulose-stabilized B8 medium for both BSCs and PSCs during long-term proliferation [98].

#### 3.2.3. Towards Serum-Free Proliferation Media for Fish Muscle Cell Progenitors

Although research on SF medium formulations for **fish muscle cells** is clearly behind the research on mammalian cells, initial studies highlight the cell type-specific effects of different GFs and demonstrate the strong responsiveness of fish muscle-derived cells to these stimuli.

**In addition to FBS and fish embryo extract**, most fish cell lines require supplementation with **additional GFs**. However, the knowledge in this area is still extremely sparse and patchy, with only a handful of suppliers offering FGF-2 and IGF-1 of fish origin. Thus, GFs and hormones typically used in fish cell culture are derived from mammalian sources (human, mouse, or rat). Identification of such crucial GFs is still a very important step towards SF media for fish cell lines.

**Embryonic fish stem cells** require basic fibroblast growth factor (FGF-2) and leukaemia inhibitory factor (LIF) [36,38,39,41], while fish muscle-derived cell lines primarily depend on FGF-2, insulin-like growth factor 1 (IGF-1), and insulin, additionally to FBS [56,75,100,101]. IGF-1 promoted the proliferation of Catmus1PFR myofibroblast precursors, but not brown bullhead catfish (*Ameiurus nebulosus*) **fibroblasts** [57] nor Atlantic mackerel (*Scomber scombrus*) **skeletal muscle** Mack1 cell line [74]. IGF-1 at a concentration of 50 nM was also shown to stimulate the proliferation of trout satellite cells under low (2%) serum conditions in DMEM and much more pronounced in F10 media [100]. In the Atlantic sturgeon larval cell line AOXlar7y, IGF-1 (50 ng/mL) was the most effective among six tested GFs at compensating serum reduction from 10% to 2%, restoring proliferation and basal metabolic activity to levels comparable to 10% FBS, while FGF-2 showed promising metabolic but limited proliferative effects [102].

A recent article reviewing the mechanisms and signaling pathways involved in the formation of muscle, fat, and other relevant tissues within the context of cultured fish [103] highlighted several factors—e.g., myostatin inhibitors, IGF mimics, and the adenylate cyclase activator forskolin—as possible enhancers of cell proliferation *in vitro*. IGF-2 could also be tested in combination with other cytokines, as medaka IGF-2 (*Oryzias latipes*), and its human ortholog, were able to support viability and pluripotency of medaka ESCs in culture media containing only basal medium [44]. The morphology of the ESCs cultured in basal medium with 200 nM medaka IGF-2 was approaching the stem cells in rich medium containing 15% FBS, medaka embryo extract, 2% of seabass serum, and 10 ng/mL of human FGF-2.

In another recent study, Dong et al. [104] explored non-animal-derived alternatives to reduce animal serum dependency by evaluating the potential of freshwater green algae *Auxenochlorella pyrenoidosa* protein extract (APE) as a partial replacement for FBS in **goldfish muscle cells** (CAM). These cells are traditionally cultured in L-15 medium supplemented with 10% FBS and 10% goldfish muscle extract. While the study did not explicitly state whether goldfish muscle extract was replaced, suggesting continued reliance on animal-derived components, the results were promising. Short- and long-term assays showed that APE supplementation significantly increased proliferation at reduced FBS levels (e.g., 5% and 2%), compared to 10% FBS controls. Notably, Myf5 expression remained consistent across all conditions suggesting that differentiation potential was maintained [104]. However, longer-term studies are necessary to confirm the sustained viability of APE as a serum replacement.

To further reduce or eliminate the remaining 5% FBS requirement, Dong et al. screened a range of additives, including antioxidants, glucocorticoids, and an ITS-based supplementation [104]. Through this screening, they identified L-ascorbic acid, a vitamin with antioxidants properties [105], and ITSE (insulin–transferrin–selenium–ethanolamine) as effective components at optimal concentrations of 10 μM and 5× (exact concentrations are not disclosed, producer: Beyotime Biotechnology), respectively. L-ascorbic acid has been previously shown to stimulate the proliferation of **pacu (*Piaractus mesopotamicus*) myoblasts** [106], while ITSE has demonstrated similar effects on mammalian muscle cells [29,30]. A combination of APE with ITSE and L-ascorbic acid is shown to effectively promote cell proliferation in the absence of FBS, with total cell numbers in the scale-up culture on day 3 almost reaching the level of 10% FBS. Another study describes a medium containing **dried Maitake mushroom extracts** used to culture **goldfish** (*Carassius auratus*) cells at comparable efficiency as the FBS containing medium [107], but it was **not successful** to support proliferation of **Mack1 cell line** [74].

Representing the most complete SF medium reported to date for fish muscle cells, Zhang et al. [108] developed an SF formulation for the *ex vivo* expansion of *Larimichthys crocea* myoblasts using a systematic screening strategy based on Plackett–Burman and Box–Behnken designs to evaluate eleven exogenous additives. The optimized medium consisted of DMEM/F12 supplemented with 5 mg/mL BSA, 20 mmol HEPES, and hydrocortisone. Viable cell numbers reached approximately 65% of those obtained in serum-containing medium, while myogenic identity was preserved. In addition, SF-cultured myoblasts showed enhanced differentiation capacity compared to serum conditions. However, the authors noted the need for safe, food-compatible alternatives to replace non-food-grade components such as hydrocortisone.

Beyond muscle-derived cell lines, Jozef et al. [45] developed the first systematically derived and fully transparent SF medium for a fish epithelial cell line (RTgill-W1), based on L-15 supplemented with ITS-X, BSA, a chemically defined lipid mixture, trace elements, biotin, vitamin B12, recombinant EGF, and hydrocortisone. Although not directly relevant to CSF production, its systematic screening strategy and identified components offer a transferable framework for SF media development in other fish cell types.

Collectively, these studies provided important insights into the development of SF medium formulations for fish cells. However, the **knowledge on fish GFs that are central to muscle development is lacking**, and our understanding of the mechanisms governing their functionality at different stages of development is rudimentary. The field remains at an early stage of development, **requiring further research** to establish reliable, scalable, and cost-effective alternatives to FBS and species-specific derived extracts for SF proliferation media of fish muscle cell precursors.

#### 3.2.4. Fibro-Adipogenic Progenitor Cells from Terrestrial Species

Only in 2022 a comprehensive review on **proliferation media for adipogenic progenitor cells** was conducted by Sugii et al. showing that in almost all the studies the medium used contained FBS in addition to diverse GFs [109].

However, recently, Hubalek et al. showed that bovine fibro-adipogenic progenitor cells (FAPs), which are suitable for cultured fat production, were able to proliferate in an SF-proliferation medium originally developed for bovine satellite cells [29], as well as in the same medium adapted for low ammonia genicity [110]. Accumulation of ammonia is a result of the metabolization of glutamine by the cells or of its spontaneous degradation in the medium. Substitution of glutamine with α-ketoglutarate, glutamate, and pyruvate in multi-passaging experiments led to a slightly higher doubling time during proliferation, with α-ketoglutarate performing best. Moreover, during both proliferation and differentiation, the ammonia concentration in adapted medium was significantly lower than in glutamine containing control and barely increasing [110]. Thus, the **study points out three different approaches to lower ammonia accumulation in SF medium for proliferation of cultured bovine fat precursors**.

#### 3.2.5. Spontaneously Immortalized Chicken Fibroblasts

A further SF medium composition for proliferation of fat cell progenitors was mentioned in Section 2. Pasitka et al. were able to adapt a **spontaneously immortalized chicken fibroblast** cell line to an albumin and SF culture medium containing only one GF and insulin (see Supplementary Table S1) [54]. Interestingly, this cell line performed even better when albumin was substituted with the following three components: 0.1% methylcellulose, 7 U/mL antioxidant solution, and 0.3 mg/mL 2-hydroxypropyl-β-cyclodextrin. This animal-component-free medium, based on DMEM/F12, was additionally supplemented with 7 ng/mL sodium selenite, 4 mM L-alanine-L-Glutamine, 5 g/L glucose, and 10 μg/mL of in-house prepared lipid mixture, and with a very low concentration of undefined GF [17]. This made a **medium price of under EUR 1/L** possible (calculated by Pasitka et al.) (see Supplementary Table S1).

#### 3.2.6. Fish Preadipocytes

The selection of an appropriate basal culture medium is critical for the efficient proliferation of **fish preadipocytes**, and DMEM was found to promote higher proliferation rates than L-15 [111]. **No SF formulations** have yet been published, nor have strategies for a FBS reduction or elimination in fish preadipocyte cultures been explored. While studies have investigated **specific molecules to enhance preadipocyte proliferation**, these approaches have exclusively used FBS-supplemented media.

Preadipocytes are routinely cultured without the addition of specific GFs in proliferation media. Some—e.g., preadipocytes from grass carp—required additional supplementation with fish serum, indicating that fish serum may contain crucial fish-specific GFs [112,113]. Cytokines such as IGF-1, insulin, and growth hormone (somatotropin) were used to improve **gilthead seabream preadipocyte proliferation** [111]. Similarly, insulin enhanced the proliferation of preadipocytes from the large yellow croaker [114], while tumor necrosis factor-alpha (TNFɑ) and docosahexaenoic acid (DHA) inhibited it. **In rainbow trout**, medium supplementation with IGF-1 stimulated preadipocyte proliferation, particularly during early phases of culture [115].

Interestingly, low concentrations of TNFɑ slightly enhanced proliferation, though higher concentrations were less effective [115]. Furthermore, DHA was shown to induce the Wnt/β-catenin signaling pathway, suppressing late-stage adipogenic differentiation and maintaining preadipocytes in an undifferentiated, potentially proliferative state [113].

Previous studies involving polyunsaturated fatty acids (PUFAs), such as eicosapentaenoic acid (EPA), DHA, and arachidonic acid (AA), have also demonstrated their capacity to enhance proliferation in skeletal VSa16 cell lines of **gilthead seabream** without exhibiting toxic effects [116]. Similarly, the proliferation of EPC-EFAD, a cell line derived from carp epithelial papilloma cells that can survive and proliferate in essential fatty acid-deficient (EFAD) medium, was stimulated by AA, EPA, and DHA in short-term cultures, while (alpha-)linolenic acids inhibited it. However, a long-term exposure to these fatty acids increased cytotoxicity, an effect that was partially mitigated by the inclusion of vitamin E in the culture medium [117].

These reports emphasize the potential **role of several components** in optimizing media composition to enhance preadipocyte proliferation, while also highlighting the **variations** in their proliferative effects **across different fish cell species**. Investigating these components and their most effective concentrations to meet species-specific requirements may present opportunities for developing SF media specifically tailored for preadipocyte proliferation. Furthermore, sourcing PUFAs from non-animal organisms, such as microalgae [118], could make them more suitable for cultured fish applications.

### 3.3. Media for Cell Differentiation and Maturation

#### 3.3.1. Myogenesis

After the proliferation phase of SCs, differentiation and maturation of myoblasts from terrestrial organisms can occur within 7–14 days *in vitro*, compared to months or years *in vivo* [119]. An irreversible cell cycle arrest of the precursor cells (myoblasts) is a prerequisite for initiating differentiation. Myogenesis proceeds with an increasing expression of muscle function genes and ends with the formation of multinucleated myofibers by fusion of myoblasts [119,120]. *In vitro*, cell cycle arrest is generally induced through **serum starvation** [121], a concept also successfully applied to fish myosatellite cells [75,100,122] and myoblasts [74,101]. Despite its effectiveness, this method still depends on animal-derived serum, although at low concentrations (e.g., 2% FBS) [29,123]. In both, terrestrial animals and fish culture, alternative protocols often substitute FBS with horse serum although it is of animal origin [74,75,101,124]. Additionally, the choice of basal medium can influence the differentiation capacity of mammalian (human) and fish satellite cells, with F10 medium yielding much lower count of MyHC-positive cells, as compared to DMEM [100].

Another important consideration when reducing and replacing serum in media is that nutrient limitation can trigger additional effects beyond the induction of differentiation. Under nutrient-deficient conditions, cells often activate autophagy, a survival mechanism that recycles intracellular components to compensate for the lack of serum, amino acids, or GFs. While autophagy initially provides protective benefits, prolonged or excessive activation can induce transition into autophagic cell death. This dual role has been well-documented, with autophagy serving as a critical adaptive response under short-term stress but becoming detrimental under sustained nutrient deprivation [125,126].

The design of **SF formulations** that drive robust differentiation of SCs independent of species has proven to be challenging [119], still, several efficient formulations have been published (see Box 4 and Supplementary Table S2 for costs comparison). Functional myotube formation using SCs extracted from adult rats was observed with an SF differentiation medium containing 10 ng/mL IGF-1 and 100 ng/mL EGF in a 1:1 mixture of Neurobasal and L-15 medium [127]. SCs isolated from bovine muscle and grown in B9 medium also differentiated efficiently into myotubes in SF-differentiation medium developed by McAleer, reaching higher fusion indices as compared to the FBS-containing medium [31]. Messmer et al. investigated transcriptomic changes in BSCs during myogenic differentiation induced by serum starvation and identified upregulation of surface receptors IGF1R, TFRC, and LPAR1 during the early phase of differentiation [128]. Supplementing SF DMEM basal medium with ligands for these receptors resulted in differentiation efficiencies comparable to serum starvation. Another—very lean—serum-free medium composition contained DMEM supplemented with TGF-β1, PDGF-BB, cytosine arabinoside, and linoleic acid, and yielded an impressive fusion index of 65.08 ± 4.97, demonstrating an improvement of 53% compared to serum-starvation method [129]. It still has to be kept in mind, that these SF formulations often use animal-derived coating proteins, such as Matrigel [128] or collagen [130], which could bind and introduce undefined cytokines of animal origin into the SF medium.

Box 4 **Differentiation media.**

⇨ Several SF media formulations are published for **myogenesis of terrestrial** species’ cell lines.
⇨ **For myogenesis of aquatic** species’ cell lines, no SF formulations are reported.
⇨ SF formulations for adipogenic differentiation of both terrestrial and aquatic species are published.
⇨ Further search for efficient differentiation inducers/ligands/stimulatory factors **suitable for the food sector** is required.

Several other supplements are known to induce myogenesis. Research on the signaling pathways that regulate myogenic differentiation unveiled that ERK1/2 inhibition induces robust myoblast differentiation and fusion of primary mouse myoblasts [131]. Although **kinase inhibitors** could provide a powerful tool to enhance efficiency of myoblast differentiation and fusion for the CM industry, cytotoxicity of kinase inhibitors must be considered as studies showed that kinase inhibitors are usually not sufficiently selective, leading to off-target effects [132,133]. Multiple studies report **nitric oxide (NO)** as a differentiation-stimulatory factor, promoting activation, renewal, and differentiation of satellite cells and ESCs [134,135]. In addition, both **mechanical and electrical stimulations** are well documented to improve the myogenic differentiation of muscle stem cells and the contractile force thereof [136,137,138].

The development of an SF medium for the myogenic differentiation of fish muscle cells has not been reported in the literature yet, but several ingredients that could induce differentiation have been identified. **Retinoic acid** at varying concentrations (from μM to mM) has been shown to promote differentiation in fish embryonic stem cells (ESCs) [36,39,40,41,139,140]. Compounds known to enhance myogenesis in fish stem cells—including FGF-2, GSK3β inhibitors, calpain inhibitors, and adenylate cyclase activators—may also offer promising avenues for improving myogenic differentiation protocols ([103], see Supplementary Table S2).

Alternative approaches to induce myogenic differentiation in fish primary SCs have been tested, and the supplementation of DMEM/F12 medium with 8% FBS, 2.5 μM RepSox (a TGFβR-1/ALK5 inhibitor [141]), 5 nM LY411575 (a γ-secretase inhibitor [142]), and 0.125 μM dexamethasone (DEX, a synthetic glucocorticoid [143]) triggered the differentiation of *Larimichthys crocea* satellite cells [90]. The suitability of these components for the food sector remains to be established. For the differentiation of fibroblasts into muscle-like cells, methods that do not require a reduction of the FBS have shown promising results. In thread-sail filefish *Stephanolepis cirrhifer*, a transition from L-15 basal medium containing 10% FBS to AIM V basal medium (Thermo Fisher Scientific, Waltham, MA, USA) containing 10% FBS successfully stimulated myogenesis of fibroblast-like cells [144]. Interestingly, the same thread-sail filefish fibroblast cells could differentiate into adipocytes when cultured in L-15 with 10% heat-inactivated SeaGrow serum (EastCoast Biologics, North Berwick, ME, USA).

Although the **maturation of myofibers** has not been extensively studied, there are several documented components and conditions that promote this process, e.g., anabolic GFs including IGFs and androgen [138]. **Co-culturing** of C2C12 myotubes with fibroblasts resulted in **higher maturity** and contractility of differentiated muscle fibres [145]. Fibroblast monolayers can function as an elastic support for contracting myotubes. Further, fibroblasts secrete extracellular matrix components and GFs that may be important for the differentiation of myotubes [145]. Myogenic fusion events and the organization of muscle fibres can also be influenced by the structural properties of **scaffolds** (for details see review from [121]) and by the exerting controls mechanical stimulation [146]. For example, in a very interesting recent paper, bio-scaffolds from autoclaved vegetables provided biomimetic stiffness and tissue-sculpting structure for the expansion and differentiation of muscle and fat-cell precursors [147].

To summarize, substantial **knowledge exists** regarding the SF differentiation of mammalian muscle cells, and several effective SF differentiation media have been published (see Supplementary Table S2, Box 4 and review [146]). In contrast, **more efforts are needed** towards the development of SF differentiation media for muscle cells from aquatic animals.

#### 3.3.2. Adipogenesis

Differentiation of precursor cells into mature adipocytes that accumulate large amounts of lipids is a complex process with multiple sequentially activated transcription factors driving the process, with PPARγ being identified as the most critical transcription factor among them [148].

**Standard protocols** for mammalian cells use an adipogenic cocktail containing **insulin, glucocorticoids** (e.g., cortisol or dexamethasone (DEX), a synthetic glucocorticoid), and 1-methyl-3-isobutylxanthine (**IBMX**—a non-specific inhibitor of cyclic nucleotide phosphodiesterases, which prevents the breakdown of cyclic AMP (cAMP) and cyclic GMP (cGMP) within cells) [149,150,151]. Progenitor or stem cell types such as preadipocytes and MSCs differentiate well into mature adipocytes in presence of this standard adipogenic cocktail [130]. This cocktail together with one **PPARγ activator** was used in most of the *in vitro* differentiation studies with cells of species considered for consumption until recently [130,152]. PPARγ agonists, such as thiazolidinediones, have been reported to be also necessary for successful maturation of adipocytes that exhibit full adipogenic characteristics [153], can be leveraged by addition of oleic acid and transferrin [154], and can even trans-differentiate myoblasts into adipocyte-like cells [155].

However, some ingredients of this classical adipogenic cocktail are not food-compatible, like the synthetic molecules IBMX, classified as hazardous and harmful if swallowed [156,157] and the PPARγ agonist rosiglitazone, for which cytotoxic effects were also reported [158]. Although these compounds are required only transiently during the initial induction of adipogenesis, searching for food-compatible options will be necessary for food production processes [159]. In this regard, Katafuchi et al. provided evidence that the C-type natriuretic peptide (CNP), an endogenous adipogenesis regulator, can **substitute IBMX** in the adipogenic cocktail [160]. Replacing IBMX (0.5 mM) with CNP (10 nM) enhanced levels of adipogenesis marker genes to almost the same range compared to the IBMX-containing cocktail [160]. However, the costs for synthetic CNP peptides will elevate costs for large-scale CM production (1 mg of CNP-22 peptide costs appr. EUR 150 (Bachem); appr. EUR 1/L medium).

In another attempt to design and simplify a food-grade formulation for adipogenesis, Mitić et al. demonstrated that out of the four inducers, **insulin** and PPARγ agonist **rosiglitazone were sufficient** to trigger adipocyte differentiation [152]. In addition, low amounts of two glucocorticoid receptor activators, namely **hydrocortisone and progesterone**, were found to be important for a homogeneous cell distribution during differentiation, as well as for cell viability and spreading. Thus, Mitić et al. demonstrated that DEX and IBMX were not necessary for adipocyte differentiation of bovine stromal vascular cells. The **simplified one-step adipogenic protocol** with a reduced number of adipogenic inducers and phases was not species specific and outperformed the serum-containing medium. The percentage of positive cells and total lipid area were significantly higher in the SF differentiation conditions used by Mitić et al. This highly efficient SF and animal component-free medium, designated DMAD (Defined Medium for Adipogenic Differentiation), is based on DMEM/F-12 and efficiently **narrowed down non-food-compatible adipogenic inducers** to only rosiglitazone (see Table S3 for full composition in [152]).

Aside from adipogenic inducers, **adipogenesis requires a constant supply of free fatty acids (FFAs)** for triglyceride production and lipid droplet accumulation [161]. In experiments with bovine stromal vascular cells and conventional differentiation media supplemented with FFAs, **oleic and linoleic acid** were found to be the most efficacious for promoting lipid accumulation during maturation of adipocytes [162]. Mitić et al. included 0.1% of a chemically defined and commercially available **lipid concentrate** in their differentiation medium. Aside from a mixture of seven free fatty acids (C14 to C20, saturated and unsaturated) and cholesterol, it contains Pluronic F-68 (used to reduce foaming in stirred cultures and to protects cells from hydrodynamic damage) and Tween 80 as emulsifying and dispersing substance [152].

For a **more food-compatible strategy**, Mehta et al. proposed the use of FFAs for induction of adipogenesis in bovine precursors, as unsaturated and branched-chain fatty acids are agonists of PPAR-family transcription factors [73]. However, FFAs can have both beneficial and unfavorable effects on adipogenic differentiation, depending on the type of fatty acids, cell type, and cell differentiation status, as well as species [159]. For example, n-6 PUFAs stimulate, but n-3 PUFAs inhibit adipocyte differentiation and PPARγ *in vivo* [163]. Porcine adipocyte differentiation was increased in a concentration-related fashion by addition of oleic acid (C18:1) or linoleic acid (C18:2), but not by addition of α-linolenic acid (C18:3) to SF differentiation medium (DMEM/F12 containing 100 nM bovine insulin, 50 ng/mL hydrocortisone and 10 µg/mL transferrin) [164]. Oleic acid also induced a strong increase in the accumulation of lipid droplets in **chicken preadipocytes** isolated with the stromal-vascular fraction [165] and was found to promote adipogenic gene expression and lipid accumulation in **bovine muscle satellite cells** [69], however, all these studies included serum in their differentiation media.

The effectiveness of **oleic acid and linoleic acid** to induce *in vitro* cell adipogenesis presents a sustainable and probably more cost-efficient opportunity for large-scale cultured fat production, as these FFAs are present at high concentrations in plant-based oils such as olive and soybean oil [166,167]. The efficacy of **soybean oil** in promoting adipogenesis has been explored in clinical applications for adipose tissue engineering [159,168,169].

In case of spontaneously **immortalized chicken fibroblasts,** from the study of Pasitka et al., discussed in Section 3.2.5, the most efficient transdifferentiation inducers in the SF medium—reaching over 80% of the cell population—were 200 μM **oleic acid** combined with 12 μg/mL L-α-**phosphatidylcholine**, which is a major component of plant lecithin. This combination showed a 2.5-fold induction of PPARγ, as compared to rosiglitazone with oleic acid, and resulted in the formation of adipocyte-like cells in the absence of IBMX or rosiglitazone, delivering the **first SF food-grade differentiation medium** for CM applications [54].

The nutritional value as well as flavor and oxidative stability of CM/CSF products depend on the composition of fatty acids in adipose tissue [170]. For high-quality beef meat, like Wagyu meat, **tenderness and juiciness** are related to high levels of **oleic acid**. A high content of **polyunsaturated fatty acids**, however, makes meat prone to fast oxidation which leads to **undesirable flavors** [170,171]. In addition, the **nutritional aspect** of cultivated fat in CM/CSF products **may be adjusted** by varying concentrations of certain fatty acids in cell culture media during the differentiation process. For example, when FFA-mixtures or oleic acid only were used in adipogenesis studies with isolated **primary bovine adipose-derived stem cells**, differentiation **solely with oleic acid** in serum-containing medium resulted in cultured bovine fat which exhibited a fatty acid composition that closely resembled that of the bovine cheek fat and suet controls [170].

Besides unsaturated fatty acids, the naturally occurring **carotenoids** (β-carotene and lutein) and **retinoids** were also shown to induce preadipocyte differentiation in cells from bovine white adipose tissue [172]. Other natural components that may enhance adipogenesis include **biotin** and **pantothenate** [173,174], as well as diverse vitamins and acetate (see Table S1 in [152]). These compounds derived from natural sources may be more suitable for the food-compatible process of producing cultured adipocytes as compared to the standard adipogenic induction cocktail.

In line with protocols established for mammalian cells, the **differentiation cocktail used for fish cells** is composed of insulin, IBMX, and DEX (reviewed in [103,175]). Additionally, a lipid mixture containing oleic and linoleic acids or an adipogenic cocktail containing fatty acids (Sigma-Aldrich reagent L5146) could enhance the adipogenic differentiation of fish preadipocytes. The adipogenic potential of fatty acids regarding fish adipocytes was assessed in bone-derived MSCs from gilthead seabream, where FFA supplementation resulted in cells adopting a rounded morphology and increased lipid accumulation, particularly with **linoleic acid**. Alpha-linolenic acid, linoleic acid, and their combinations significantly upregulated the expression of fatty acid transporters FATP1 and FABP11, correlating with the enhanced lipid content of adipocytes [176].

Concerns regarding excessive fat accumulation in **farmed fish,** which could impact production levels, meat quality, consumer perception, and animal welfare [177,178], have stimulated the research on fish adipogenesis (reviewed in [161]) and identified molecules that enhance adipocyte differentiation, such as triiodothyronine (T3), biotin, pantothenic acid, ciglitazone, transferrin, and hydrocortisone (reviewed in [103,175]).

Until recently, only **one study** [179] had investigated **SF formulations for fish adipocyte differentiation**, notably also excluding IBMX. For **stromal-vascular cells** of **red sea bream** adipose tissue, a differentiation medium consisting of DMEM/F12 (1:1) containing 0.042 mg/L linoleic acid, supplemented with 65 mM NaCl, 50 μg/mL transferrin, 5 ng/mL sodium selenite, and 50 ng/mL hydrocortisone, has been used to induce terminal adipocyte differentiation [179]. In the absence of FBS, hydrocortisone was found to be essential. The authors also reported that insulin significantly enhanced adipocyte differentiation and facilitated lipid accumulation in a concentration-dependent manner. While T3 alone did not impact differentiation, it did increase differentiation-linked lipoprotein lipase (LPL) gene expression when combined with insulin. In contrast, fat-soluble vitamins had no effects on adipocyte differentiation. Other studies confirmed the role of insulin in stimulating adipocyte differentiation in both gilthead seabream [111] and large yellow croaker [114]. Interestingly, IGF-1 was identified as a more effective inducer of differentiation compared to insulin in gilthead seabream [111].

More recently, two studies from the same group further explored strategies to reduce reliance on FBS for adipogenic differentiation in *Larimichthys crocea*. Wei et al. [180] demonstrated that adipogenic transdifferentiation of muscle satellite cells could be achieved by replacing FBS with horse serum (10%) in combination with oleic acid and conventional inducers (dexamethasone, insulin, IBMX, rosiglitazone, and indomethacin); notably, FBS could not substitute horse serum in this context, highlighting that serum source critically determines adipogenic induction capacity in fish cells. Moving towards a fully serum-free approach, Zhou et al. [181] developed a defined serum-free induction platform for adipose-derived stem cells from the same species, combining a plant-derived small molecule cocktail—bavachinin and dehydroabietic acid (BD)—with a serum substitute (SS) comprising growth factors (bFGF, EGF, IGF1), antioxidants (sodium selenite, glutathione, and ascorbic acid), and carrier proteins (recombinant human serum albumin and transferrin). The SS + BD combination effectively activated key adipogenic regulators (PPARγ, C/EBPα, LPIN1) and supported lipid accumulation comparable to FBS-supplemented conditions, with further improvement observed under three-dimensional culture on soft gelatin-based microcarriers (10 kPa).

While current progress in serum- and IBMX-free media for fish adipogenesis is promising, further research is needed to **assess gene expression profiles** and **signaling pathways** involved in adipocyte precursor proliferation and differentiation. These studies are critical for identifying species-specific needs and developing targeted supplements to optimize (pre)adipocyte culture. For instance, gilthead seabream MSCs from adipose tissue and bone were shown to differentiate into adipocyte-like cells, with distinct lineage-specific regulation of adipogenic gene expression [182]. This highlights the influence of cell origin on adipogenic pathways and the need for tailored culture conditions. The study also identified key genes regulating adipogenesis in gilthead seabream, providing valuable insights into lipid storage regulation in fish [182]. These findings underscore the importance of further investigations into species- and cell-specific mechanisms to improve media formulations and cell culture techniques.

To summarize, in both CM and CSF areas, efficient SF adipogenesis-promoting media formulations are published, including those only using food-grade components (see Supplementary Table S3 for contents and cost comparison, and Box 4). Additional research is needed to cover further cell types and species.

## 4. Strategies to Reduce Costs of Cell Culture Media

A critical prerequisite to produce CM/CSF is an adequate cell culture medium that provides all essential components required for cells to grow, replicate, and differentiate. Approximately 50% to 90% of the material costs for cell-based food production refer to the cell culture medium [94,183,184]. Garrison et al. estimated that 1 kg CM produced in a large-scale setting will cost USD 63/kg (around EUR 56/kg) assuming that future technologies will reduce costs for media [185]. For reference, the wholesale price of lean pork was EUR 8.12/kg and lean beef was EUR 14.12/kg in 2024 [186]. CM manufacturers [184] as well as researchers [187] anticipate that the medium price has to fall to **EUR 1/Liter** to reach price parity with conventional meat. Therefore, the cell-cultured meat and seafood industry requires game-changing innovations to facilitate an economically high-volume production capacity, which are discussed in this section (see Box 5 and Table 2).

Box 5 **Strategies to lower cell culture media costs.**

⇨ **Substitution of FBS with hydrolysates: only partial replacement** possible for CM/CSF cell lines.
⇨ **Recombinant GFs** and **cell line adaptation** are effective alternatives to FBS.
⇨ **Increasing stability and biological activity of GFs** in culture media significantly cut costs (for the list see Table 5).
⇨ Low-cost food-grade **HSA alternatives** are available.
⇨ Hydrolysates are a valid part of an **alternative to a chemically defined basal medium.**
⇨ Circular cell culture can further **lower the medium price and reduce waste generation.**

Cost reductions can be achieved by altering the medium formulation or by using cheaper, food grade alternatives, e.g., by-products of agar or food industry like plant or yeast extracts, strategies which have been described in recent reviews [61,88,188]. Some companies are already producing and selling food-grade media at industrial scale—e.g., Nutreco (The Netherlands), Multus (UK). Using this strategy, Mosa Meat reported to have reduced its media costs by the factor of 88 [189]. Still, caution must be taken, as food/feed grade products may have performance issues compared to pharma-grade alternatives due to lower purity. This includes an increased potential for contaminants, as well as having lower efficiencies in cell performance characteristics due to differences in processing/storage/etc. of food-grade media compounds, which must be monitored.

The constituents of FBS, such as GFs and albumins, have been identified as the most dominant cost drivers for the media [94,184,190]. However, they are required for most of the CM/CSF relevant cell lines. Basal media offers further opportunities to reduce media production costs and simplify the logistics of sourcing. In the following sections, we will therefore focus on strategies to reduce the associated costs.

### 4.1. Efforts to Substitute FBS with Hydrolysates

#### 4.1.1. Partial Substitution of FBS by Plant Hydrolysates

For cell lines not applicable in the CM/CSF area, research data indicated that protein-rich plant hydrolysates, such as those derived from soy, wheat, and rice, are non-toxic and can fully substitute FBS. Protein hydrolysates improved cell viability, proliferation rate, and resistance against shear stress for CHO, HEK, VERO, BHK as well as insect cell lines [191,192,193].

The effect of hydrolysates seemed to be not only nutritional, which gave rise to a theory that higher-MW oligopeptides present in plant hydrolysates could act as growth or survival factors.

The same effect was also anticipated for CM-relevant cell lines, but as for now, only partial substitution of FBS was demonstrated, and is discussed in detail by Flaibam et al. [194] and Mi et al. [195].

**Table 2:** Strategies for the reduction of the costs associated with cell production and differentiation for cell-based food, discussed in this review.

Approach	Components	Costs and Comments	Food-Compatibility Assessment	References
**General approaches**				
**Switch from pharma grade to food grade compounds**	Glucose, amino acids	Glucose (EUR 1/kg instead of EUR 90/kg); Glutamine (EUR 40/kg versus EUR 280/kg).	Good	[61]
**Reduce concentration of proliferation-promoting proteins**	FGF-2, TGF-ß, insulin, transferrin	Reduction by appr. 90% of the costs for in-house prepared E8; possible negative effects—determination of appropriate concentration for each factor and cell line/species required.	Good	[94]
	Proliferation-promoting proteins in B8	A 3:1 dilution of B8 in DMEM-F12 in presence of stabilizers like methylcellulose or alanine sustained cells viability for four days.	Good	[98]
**Appropriate choice of GFs or orthologs**	Human recombinant IL-6	Cost reduction by 80%, increased proliferation of bovine myoblasts.	Unclear	[29]
	Atlantic salmon FGF-2	Best FGF-2 ortholog to promote bovine SC proliferation. Best results with 25 ng/mL. B8 contains 40 ng/mL recombinant FGF-2.	Unclear	[196]
**GF alternatives**	Peptides, peptoids, small molecules	Lower production costs, higher stability, but unclear safety and regulatory status.	Unclear	[197]
**Spontaneous cell line adaptation**	Adhesion→suspension, lower GF requirements	Allows adaptation of continuously proliferating cell lines to lower GF concentrations (up to 10–20 times lower medium price) and/or to proliferation as single-cell suspensions (40-fold higher cell densities).	Unclear	[53,54]
**Hydrolysates**	Glucose, vitamins, amino acids, minerals	Plant extracts can replace nutrients in media, allowing lower FBS concentration.	Good for traditional food plants	[198,199,200]
		Microalgae can replace nutrients in media, including glucose, allowing lower FBS concentration.	Good upon safety assessment	[201,202,203,204]
		Microbial cells can replace nutrients in media but also substitute HSA after adaptation period.	Unclear	[192,205,206]
**Recycling of waste media**	Glucose, vitamins, amino acids, minerals, lactate	Microalgae *Chlorococcum littorale* and RL34 hepatocytes allowed at least two full media cycles, after replenishing GFs and media components using microorganisms. Genetically modified cyanobacteria reduced lactate assimilation.	Unclear	[207,208,209]
**Protein engineering**				
**Increase of GF thermal stability by mutations**	FGF-1	Two single mutations led to increased proteolytic stability, but did not increase thermostability.	Unclear	[210]
	hFGF-1	Mutating three sites increased stability.	Unclear	[211,212,213]
	FGF-2	Triple and quintuple mutations improved stability and caused 10-fold prolonged biological activity.	Unclear	[214,215]
**Increase affinity of GF for heparin**	hFGF-1	Engineering of hFGF-1 heparin binding pocket increased affinity for heparin, but cell proliferation activity of mutated variant was reduced. Heparin is of animal origin and this may be problematic.	Unclear	[216]
**Heparin-independent GF analogues**	FGF-1	FGF-1 variants with increased stability were less dependent on heparin.	Unclear	[212]
	FGF-2	Similar observations for FGF-2 variants as for FGF-1.	Unclear	[217]
	FGF-2	GF-dimerization by chemical conjugation of two mutated FGF-2 preserved its native conformation and reduced heparin dependence.	Unclear	[218]
**Modify binding affinities of GF to receptors**	EGF	Site-directed mutagenesis resulted in 30-fold higher affinity of EGF for its receptor. This strategy may lead to rapid receptor internalization or unknown modified signaling effects.	Unclear	[219,220]
**Minimal receptor agonists**	HGF	Two copies of the kringle 1 (K1) domain in tandem orientation displayed biological activity like native HGF. K1K1 is one fifth of the full-length protein, thus decreasing costs for recombinant GF production.	Unclear	[221]
**Immobilization of GFs**				
**Physical immobilization**	bFGF	Multilayered biodegradable microspheres (heparin-conjugated alginate covered by chitosan and dextran-sulfate). Sustained release for two weeks.	Good	[222]
	FGF-1	Multilayered alginate microcapsules coated with poly-L ornithine membrane. Sustained release for three days, but only co-encapsulation with heparin retained FGF-1 active.	Good	[223]
	TGF-b1	Chitosan microspheres; encapsulation in presence of tripolyphosphates; sustained release for 7 days	Good	[224]
	FGF-2	Hydrogel carrier made of chitosan and fucoidan; gradual release of FGF-2 upon biodegradation of hydrogel.	Good	[225]
	FGF-2	Microspheres made of collagen and alginate polymers; sustained release.	Good	[226]
	FGF-2	PLGA microspheres; sustained release for 72 h. Synthetic polymer! Food grade?	Unclear	[227]
**Covalent binding to surfaces**		Achieve gradual release, but requires complex methodologies, thus resulting in increased costs.	Good	[228,229]
**Addition of stabilizers**	FGF-2	High concentrations of methylcellulose, alanine and HSA, etc., massively increased GF stability.	Good	[230,231]
	GFs in B8	Rapeseed protein isolates successfully substitute HSA in B8 medium.	Good	[190]
	GFs in B8 and B9	Methylcellulose extended greatly the stability of GFs in DPBS.	Good	[98]

Thus, plant-derived hydrolysates can enhance cell proliferation by contributing nutritional value, but they can only **partially replace FBS** in CM/CSF cell culture media. The limited possibility of plant extracts to substitute FBS may be attributed to the absence of essential GFs, which are necessary for the proliferation of primary or any cell lines, not adapted to protein-free media. On the other hand, some peptides are known to promote cell proliferation and differentiation to some extent, e.g., via G-protein-coupled receptors or mTOR pathway (see the review [195] for details). It appears that GFs could not be replaced by the protein/peptide mixture derived from the hydrolysis of the plant material, although significant reduction of FBS was possible for multiple cell lines [194,195,232]. However, plant extracts provide a diverse source of nutrients like amino acids and peptides, carbohydrates, lipids, and minerals [233] which could contribute to the basic ingredients of cell culture media formulations.

#### 4.1.2. Partial Substitution of FBS by Microalgae Hydrolysates

The potential of microalgae extracts **to lower FBS** concentrations in cell culture medium has been proved using goldfish *Carassius auratus* muscle cells [104]. Supplementation of Leibovitz’s L-15 medium with ***A. pyrenoidosa* extract (1 mg/mL)** and 5% FBS significantly promoted the proliferation of fish muscle cells, which could be continually cultured for four passages, as compared to 10% FBS control [104]. **Low serum conditions** in combination with the use of extracts from the microalgae ***C. vulgaris*** as a cell-culture additive also successfully promoted the proliferation of CHO cells and MSCs for up to 21 days [234,235]. Cell proliferation with a maximum increase in cell viabilities was observed at algae extract concentration of 0.001 g/L. These results highlight that microalgae extracts can act as a functional supplement enhancing the nutritional properties of mammalian culture media.

An **SF medium** supplemented with algae extract was studied by Yamanaka et al. [203]. First the authors used ***C. vulgaris*** extracts in addition to an inorganic salt solution as nutrient source for maintaining rat liver epithelial RL34 cells which secreted **IGF-1 and IGF-2**. The culture supernatant containing these GFs supplemented with *C. vulgaris* extracts was then provided to bovine myoblasts as cell culture medium. Compared to FBS-containing media, cell numbers of bovine myoblasts cultured in such medium were reduced indicating that this SF medium with GFs IGF-1 and IGF-2 and *C. vulgaris* extract could replace only partly FBS [203]. Additionally, the combination of *C. vulgaris* extract with GFs **FGF and TGF plus insulin** as supplement for SF DMEM was not sufficient to reach proliferation rates comparable to media containing 10% FBS, as shown for bovine fibroblasts [236]. In June 2021, the South Korean company SeaWith published an announcement stating that they are using microalgae as an FBS replacement for manufacturing CM (https://seawith.net/news/media, accessed on 9 June 2026). Media details, however, are not available.

#### 4.1.3. Partial Substitution of FBS by Yeast Hydrolysates

Yeast-based hydrolysates have been widely used as supplements for CHO cells cultured in an SF medium (see review by Ho et al. [233]). **Yeast extracts** have the potential to partly **replace serum** in mammalian cell culture media used for CM production. The proliferation of bovine primary skeletal muscle cells in SF conditions, in the short-term (48 h), could be partly restored by the addition of a commercially available yeast extract [205]. Yeast extract-enhanced cell metabolic activity by more than 100% and compared to the control without FBS, cell proliferation raised by almost 50%. However, this effect was concentration-dependent with an upper limit of 1 g/L yeast extract. Higher concentrations showed cytotoxic effects to the cells. Furthermore, the authors observed that especially hydrolysates rich in **peptides** with approximately **2–15 amino acids** in length enhanced cell proliferation [205]. Additionally, as discussed in the Section 4.4.6, 100 mg/L yeast hydrolysate has significantly improved proliferation of Mack1 fish cell line in 2.5% FBS-containing medium [74].

In the light of the findings that the addition of yeast hydrolysates to animal cells *in vitro* does not show adverse effects and is even able to support short-term cell proliferation, they can potentially be used as a part of an alternative basal medium (discussed in 4.5.3). Further, a complementing strategy could work for long-term proliferation, as required in CM/CSF production. A paper of Lei et al. [237] has demonstrated that the addition of sterile filtrated wet yeast lysate of a *S. cerevisiae* strain, simultaneously expressing four cytokines (human bFGF, EGF, PDGF-BB, and LR3-IGF-1), was able to promote cell proliferation of porcine SCs for 3 days in presence of 5% FBS to the same level as 10% FBS. Although the non-hydrolyzed yeast cell lysate with the above-mentioned recombinant GF combination was also not able to completely substitute FBS, the application of GFs as part of yeast lysates—for example, in the same production pipeline, but prior to hydrolysis to prevent GF break down—can be used to avoid the need of GF purification, which can make up to 50% of the GF production cost.

### 4.2. Reducing the Concentration of Proliferation Promoting Proteins

**A reduction of the concentration of four recombinant proliferation promoting proteins (insulin, transferrin, FGF-2, and TGF-β)** by one tenth of their current level resulted in USD 41 (around EUR 36) per L of medium, a price which accounts for only 11% of the costs estimated for an in-house prepared E8 [94]. Low-serum adaptation is routine, and the same principle could be applied for individual GFs. However, a reduced concentration of these supplements may have negative effects on cell proliferation and maintenance. In this regard, Stout et al. have demonstrated that in the long-term culture of primary non-adapted BSCs, the attempt to lower FGF-2 concentration in B8-based medium led to a significantly lower cell number [31]. On the other hand, they have shown that concentration reduction of proliferation promoting proteins could still be a reasonable strategy if combined with stabilization approaches. This was confirmed in the paper by Schenzle et al., where the dilution to 70% of the original concentrations of B8 and B9 media could be rescued by addition of stabilizers, namely HSA or, alternatively, methylcellulose (MC) with starch from corn [98]. However, the use of HSA among all stabilizers must be avoided due to its lack of food compatibility and low sustainability. Please see Section 4.4.5 and Section 4.4.6 for the published HSA alternatives, meeting these criteria.

Another strategy to lower the GF concentration is the cell line adaptation, discussed on an example of immortalized chicken fibroblasts in Section 3.2.5. and on fish cell lines in Section 2.

### 4.3. Appropriate Choice of Growth Factors or Orthologs

GFs are proteins secreted by cells to communicate with each other. They are essential supplements for SF cell culture media as they mimic endogenous signaling pathways to promote proliferation, and differentiation of cells. The choice of GFs used in CM/CSF production is dependent on the animal species, cell type, and culture process [238] and is discussed in Section 3.2.

The available supply of recombinant GFs from a variety of species provides a substantial pool of GFs that can be analyzed for its effect on the proliferation of cells *in vitro*. When testing various GF orthologs for their ability to promote bovine SCs proliferation, Venkatesan et al. showed that Atlantic salmon FGF-2 was the most potent among all the FGF-2 orthologs tested [196]. Highest effect was observed at 25 ng/mL, but this decreased with higher concentrations. The most effective GF for bovine SCs was PDGF-BB from Eagle; however, at a concentration of 100 ng/mL. In contrast, human Tgf-β1, showed no improvement at all concentrations tested compared to FBS controls [196]. In another study, replacing bovine recombinant IL-6 with human recombinant IL-6 not only led to a staggering cost reduction of 80% but also significantly increased the proliferation of bovine myoblasts [29].

Most of the GFs used for fish cell cultures are recombinant and based on mammalian GF sequences. However, as mentioned in the Section 2, protein sequence identity of GFs between fish and mammals, as well as between different fish species, is sometimes quite low [44]. Thus, effective species-cross-reactive GFs in case of fish cell lines are less probable. As discussed in more detail in Section 3.2.3, only a handful of providers produce fish GFs, although the situation is gradually improving, including more research on species-specific GFs being done.

Thus, identifying the most effective GF ortholog and determining its minimal concentration or finding appropriate GF combinations for individual species and cell types will be a prerequisite to enable long-term cell proliferation and to maintain cells in an FBS-free environment for an economic *in vitro* propagation and differentiation of non-adapted cells. However, it should be kept in mind that the industry is currently leaning towards **using species-specific GFs** due to regulatory requirements, although it is **not always the most efficient choice** (https://gfi.org/resource/cultivated-meat-media-growth-factor-survey/#species-specific-growth-factors, accessed on 9 June 2026) (see also Section 5 about Regulatory Submissions).

### 4.4. Addressing the Issue of Growth Factors’ High Costs

GFs are key components of SF medium used for cellular agriculture applications of non-adapted mammalian cells. Many of them require post-translational modifications for their functionality which makes the production of recombinant GFs challenging and expensive [196]. Moreover, GFs are inherently unstable molecules because they are biologically intended to act transiently as short-term signals [230]. Hence, sequential multiple administrations and/or supraphysiological concentrations are required to sustain an effective concentration of GFs in media, resulting in higher costs and possibly adverse effects [220]. Thus, technological approaches with the aim to significantly improve GF stability and bioactivity are crucial factors for reducing GF concentrations and costs for CM/SCF production. This is also reflected in the recent surveys, showing high interest of manufacturers and CM producers in modified GFs (https://gfi.org/resource/cultivated-meat-media-growth-factor-survey/#modifications, accessed on 9 June 2026). In this section we will discuss approaches aiming at GFs with (1) **higher stability**—e.g., through specific point mutations, immobilizations, or addition of stabilizers, and (2) **higher potency** through enhanced binding affinity to appropriate cell receptors(s). Other approaches (3) to lower the medium costs by substituting GFs with **peptides** from plant hydrolysates or synthetic peptides, or with **small molecules** will be also analyzed. For a comparative summary of diverse expression hosts for GF production in CM applications please see the review by Mi et al. [239].

#### 4.4.1. Increase Growth Factors’ Stability Through Mutations

A characteristic feature of the structurally related and highly conserved members of the FGF family proteins is their low thermodynamic stability and relatively short half-life *in vivo*. At physiological pH and temperature almost 50% of the **FGF-1** protein is unfolded within less than an hour and therefore prone to proteolytic degradation [211]. Prolonged biological activity of FGFs can be achieved by increasing their proteolytic resistance. Proteolytic cleavages occurred mainly within the C-terminal region of the naturally occurring (non-engineered) FGF-1, pointing on its significant role in GF degradation [210]. Based on bioinformatic analysis, two single mutations (C117P, K118V) were introduced within the C-terminal region and combined in a double mutant. This led to a rigidification of the FGF-1 segment, which is highly sensitive to proteases, reducing *in vitro* susceptibility to trypsin and chymotrypsin degradation significantly, as compared to the non-engineered FGF-1. Thermostability, however, was not improved by introducing these mutations [210].

Significant efforts have been made to clarify the function and mechanism of action of heparin binding to GFs, which appears to strongly improve GFs’ stability and affinity for receptors. Heparin-binding sites are prevalent in the GFs utilized in the CM industry, and are present e.g., in FGF-2, HGF, IL-6, PDGF, VEGF [240]. Heparin, which is a stabilizing glycosaminoglycan that is commonly found in the extracellular matrix (ECM) of mammalian cells, binds to a cluster of positively charged amino acid residues of FGFs, which are located at the C-terminal end of the molecules [241]. Sulfate-rich polymers like heparin and other—plant, animal or synthetic—heparinoids [242] can help to preserve GF activity by extending their half-life at 37 °C [243]. **However, the use of heparin** from animal sources or of its synthetic analogues as an additive in cell culture media may be problematic for CM/CSF production. It is also susceptible to batch-to-batch variability and contamination, as it is isolated from animal tissues. Fractionated heparin is associated with fewer side effects, but the process of fractionation is costly [244]. Alternatively, **plant heparinoids** could be investigated for applications in cellular agriculture [242,243].

The stability of **hFGF-1** could be increased by selective engineering of the heparin binding pocket via charge reversal mutations, but the cell proliferation activity of the resulting hFGF-1 mutant was reduced compared to the wild type hFGF-1 [216]. The weak thermostability of wild type hFGF-1, which loses its activity after 6 h at 37 °C even in presence of heparin, could be significantly improved by point mutations at amino acids Q40, S47, and H93 [212,213]. By mutating these three sites, FGF-1 became significantly thermo-stabilized, while its activity was maintained. This increased stability could compensate for reduced affinity to heparin when in addition the heparin binding site K112 was mutated to asparagine [211].

Based on sequence alignments of stabilized FGF1 mutant sequences and non-engineered **FGF-2**, triple [214] and quintuple [215] FGF-2 mutants with significantly improved stability were generated. Further, a hyperstable nine-point mutant named FGF2-G3 (or FGF2-STAB2) was constructed by employing focused directed evolution and combining individual stabilizing mutations published elsewhere [217,245]. In this mutant, functionally important positions for the heparin binding were left unchanged. The melting temperature of this mutated FGF-2 variant increased by 19 °C and its functional half-life at 37 °C improved from 10 h to more than 20 days *in vitro* [245], which might allow both the reduced concentration and reduced frequency of spiking. This variant (FGF2-G3 or FGF2-STAB2) is commercially available from several distributors.

In their stability studies using **FGF-2** from human and zebrafish, Chen et al. found that both GFs were not degraded after 24 h incubation at 37 °C but aggregation caused a loss of FGF-2 biological activity [213]. Such aggregation events were prevented by **heparin-binding** and point mutations in the conserved heparin binding sites of FGF-family proteins. Interaction with heparin restored the optimal FGF protein folding and increased its thermostability [212,213].

Generally, FGF-1 variants with increased stability were shown to be less dependent on heparin binding for receptor binding and signaling [212], demonstrating the effectiveness of this strategy. Similar observations were made regarding FGF2-STAB variants with improved thermal stability. Compared to non-engineered FGF-2, FGF2-STABs showed higher efficiency in promoting proliferation and lower affinity to heparin [217]. Thus, protein engineering resulting in strong and powerful stabilizing effects may abolish heparin dependence of FGF proteins. Therefore, the development **of soluble, heparin independent GF analogues** with improved stability by protein engineering is the preferred strategy for obtaining stable and bioactive variants of most crucial GFs used in CM/CSF area. This approach was successfully applied for HGF [221], leading to a more potent minimal variant, as well as for VEGF [246]. However, the regulatory status of such stabilized GF mutants is still to be determined.

#### 4.4.2. Strengthening Signaling Response by Modifying Binding Affinities of Growth Factors to Receptors

Modifying **binding affinities** between GFs and their receptors may influence the magnitude and duration of the signaling response. A prolonged cellular response to GFs at lower concentrations would also contribute to cost reduction for culture media. The affinity of EGF for its receptor was up to 30-fold higher after amino acid residues Ile 38, Glu 51 and Leu 52 in EGF were changed by site-directed mutagenesis [219].

**FGF-2 dimerization** is essential for the formation of an active ligand–receptor complex. As such, a covalent linkage of two FGF-2 molecules provides another strategy to improve the biological activity and stability of GFs [218]. To engineer FGF-2 dimers, mutants with single surface-exposed reactive cysteine for the chemical conjugation with PEG were generated. All dimeric proteins showed enhanced mitogenic potential, as compared to FGF-2 monomer; the most significant differences were detected at 10 ng/mL protein concentration [218]. Dimeric FGF-2 variants did not show any tendency to aggregate and—in contrast to non-engineered FGF-2—the amount of functional dimers was not significantly altered after 48 h in the presence of cells [218]. Thus, FGF-2 dimerization provides the possibility to reduce the need for heparin as well as costs as dimeric proteins possess increased stability in cell culture medium.

A similar strategy was applied to improve the limited stability of native hepatocyte growth factor/scatter factor (**HGF**). Non-engineered HGF is comprised of two-chains (α/β). The 64 kDa α-chain contains four kringle (K) domains and the high affinity binding site for the MET receptor. The β-chain (34 kD) contains a secondary binding site for MET [247]. A **minimal receptor agonist** termed K1K1 consisting of two copies of the kringle 1 domain of HGF in tandem orientation was designed and tested both *in vitro* and *in vivo*. K1K1 was stable and displayed biological activity which was equivalent or superior compared with native HGF [221].

For GF engineering approaches, the design of short GF variants mimicking full length GF activities would provide a powerful tool **to reduce costs** for recombinant expression of proliferation-promoting proteins required for media supplementation for CM/CSF production. This strategy, however, requires extensive information about binding mechanisms of GFs to their receptors as well as detailed structural data regarding receptor-activating configurations. The creation of realistic models to simulate distinct receptor-ligand interactions is a prerequisite for predicting minimal GF variants or mimetics with increased bioactivities and stabilities.

#### 4.4.3. Immobilization of Growth Factors

The use of **immobilized GFs (iGFs)** may be a promising approach for CM/CSF production as it gives the possibility to achieve a higher GF stability and a sustained release, which may considerably reduce the need for multiple doses, facilitate recycling of GFs and consequently decrease associated costs for cell cultures.

**Physical immobilization** is technically the simplest method to immobilize GFs, commonly achieved by adding GFs into a polymer matrix before its gelatinization [228]. This and other GF immobilization strategies are extensively reviewed elsewhere [228]. Several natural and inexpensive biopolymers can be used for microencapsulation and regarding food-safety, polymers of plant origin like alginate or starch are of special interest [248]. Below we present examples of GF immobilization in food-relevant matrixes, showing sustained release.

**Multilayered alginate** microcapsules coated with a permeable poly-L-ornithine (PLO) membrane were used for the encapsulation of FGF-1 [223]. Release kinetics showed a burst release of FGF-1 within the initial 5 h, followed by a low-concentration continuous release for up to thirty days. However, FGF-1 released from microbeads was active only when heparin was co-encapsulated in the outer layer [223].

In contrast, alginate microspheres **failed to release Tgf-β1** [224], possibly because of the interaction between the carboxyl groups of alginate and the amine groups of the GF. Therefore, Tgf-β1 was loaded in **chitosan microspheres,** which were incorporated in porous chitosan scaffolds. **Chitosan** has been reported to stimulate and protect the activity of GFs [243,249] and it possesses a cationic nature resulting from primary amine groups. Chitosan microspheres were loaded with Tgf-β1 by the emulsion-ionic crosslinking method [224]. The release curve showed a strong release within the first 3 days, followed by a slow sustained release of Tgf-β1 over several days [224].

A 1:2 (wt) mixture of **chitosan and fucoidan**—a sulfated polysaccharide derived from brown seaweeds—was used to encapsulate FGF-2 for controlled GF release [225]. FGF-2 molecules were incorporated in the chitosan/fucoidan micro complex-hydrogel, which substantially prolonged the biological half-life time of FGF-2. Moreover, a gradual release of FGF-2 from the hydrogel was determined *in vivo* [225].

Microspheres composed of readily degradable **collagen** with more slowly degradable, mesh-forming **alginate polymers** were used to study sustained release of FGF-2 in relation to varying amounts of collagen [226]. *In vivo* experiments using zebrafish embryos showed that FGF-2 released from such microspheres induced angiogenesis [226]. Collagen, which is usually extracted from animal tissues, is now available from recombinant biosynthesis [250], though at a high price.

Aside from natural polymers, synthetic biocompatible and biodegradable polymers such as **polylactic glycolic acid (PLGA)** have also been described for sustained release formulations of GFs as shown for FGF-2 [227]. PLGA is a biodegradable linear co-polymer composed of lactic and glycolic acid, which has been approved for clinical use in humans by the American Food and Drug Administration [251]. However, such biopolymers used for medical applications may not become approved for food industry.

The **impact of GF immobilization** on reducing the overall manufacturing cost for CM/CSF will depend on a number of parameters, e.g., the cost for (recombinant) GFs, the selected immobilization method including the costs for biomaterials used, or immobilization surfaces. Loading efficiency, release kinetics, stability, and biological activity of each GF will have to be tested and evaluated for every immobilization technique together with cell lines/species used for CM/CSF production processes. These are main challenges that lies ahead in the development of engineered and immobilized GFs and will require further research in this field.

#### 4.4.4. Growth Factor Alternatives

As GFs account for a significant portion of the total media costs in most media compositions, there has been an ongoing effort to substitute them with alternative molecules that are more cost-effective. The substitution of the GFs with defined peptides, peptoids, or small molecules, which mimic GFs and can specifically activate respective receptors, might be a usable strategy. They are generally more stable, less susceptible to proteolysis and cheaper in production [197]. For example, Kenichiro Ito et al. presents an HGF-mimicking peptide, which at about two orders higher concentrations than wild type HGF was able to achieve the same level of Met-selective activation of the Erk signaling pathway [252]. Peptide and peptiod mimetics for other crucial GFs used in cell cultures have also been developed, for example for FGF-2 and PDGF-B, which, together with multiple HGF mimetics are extensively reviewed elsewhere [197]. Such peptides offer notable advantages, including reduced immunogenicity and lower production price compared to proteins, along with the potential for enhanced interaction with larger receptor surfaces in comparison to small molecules [253,254].

Finally, GFs can be substituted with small molecules, which efficiently activate respective receptors. Researchers, connected to the German CM start-up “The Cultivated B” have recently identified a guanylhydrazone-based molecule (TCB-32) via structure-based virtual screening on the orthosteric binding site of FGFR1. They have then further optimized it to yield three highly potent agonists of the FGF receptor [255]. Furthermore, Believer Meats identified small molecules—agonists of FGF-2 [256]. This demonstrates interest of CM industry for such compounds. Other small molecules mimicking FGF-2 and HGF were also reported, though for pharmaceutical applications [197].

However, a **significant challenge** in utilizing such GF mimetics is the **lack of their safety assessments for food applications**, as most of them were originally developed for medical purposes. An additional challenge could be to find natural products/molecules that act similarly to the mimetics described above. Very promising in this regard was the recent study with forskolin—a natural bioactive diterpene extracted from the roots of the Indian coleus plant (*Coleus forskohlii*)—which was shown to boost the proliferation of BSCs in Beefy-9 medium over six passages, yielding 1.9 times more BSCs [257]. This effect was further enhanced in the combination with p38 inhibitor SB202190, which was previously shown to preserve the PAX7 expression and improve proliferation of the BSCs [65,258].

#### 4.4.5. Extending the Activity of Growth Factors by Addition of HSA and Other Known Stabilizers

From a cost perspective, the addition of excipients for stabilizing GFs in media would be the preferred approach, which is also relatively simple to implement in bioprocesses. Substances like salts, sugars, polymers, proteins, and amino acids may be used for this purpose [230]. In their attempt to preserve functional FGF-2 molecules in **aqueous solutions**, Benington et al. tested a number of approved pharmaceutical excipients—alone or in combinations—in order to identify potential stabilizers for soluble FGF-2 [231]. With this systematic approach, the addition of 0.5% *w/v* methylcellulose (MC)—an emulsifier used in food industry also known as E461—strongly improved the protective effect of the alanine (20 mM) and human serum albumin (HSA) (1 mg/mL) on FGF-2 against rapid thermal degradation at 37 °C. Insignificant losses of FGF-2 contents after 2 h at 37 °C were determined under these conditions. The FGF-2 formulation with 0.5% *w/v* MC and 20 mM alanine was also stable when incubation time at 37 °C was prolonged to 8 h, retaining 97% of FGF-2) [231].

Polymers like **methylcellulose** form hydrogels which increase molecular density and viscosity of aqueous solutions and prevent aggregation of proteins like GFs. This effect was more pronounced at low MC concentrations [231]. **Amino acids** such as alanine and glycine, also used as cryoprotectants for protein formulations, contribute to protein stability through weak electrostatic interactions [259]. Benington et al. reported that alanine, but not glycine, met the criteria of an effective stabilizer for FGF-2. Like for MC, lower concentrations of alanine were significantly more effective at stabilizing FGF-2 at higher temperatures [231].

**Albumins, which are major constituents of mammalian serum,** are known to stabilize the structure of other proteins through ionic, electrostatic, and hydrophobic interactions and could be preferred over amino acids due to their metabolic inertness [230]. This stabilizing effect on GFs was most probably the reason for the clearly improved proliferation of bovine satellite cells (BSCs) in B8-medium supplemented with **recombinant HSA** (800 μg/mL) [31]. The use of stabilizers, like **recombinant albumin**, however, would result in a substantial increase in medium costs.

In search of solutions to this problem, several groups tested known but also previously unreported stabilizers for their ability to substitute this function of HSA in the cell culture. In the interest of minimizing costs for cell culture media, Schenzle et al. tested the addition of different concentrations of low-price stabilizers for extending the activity of GFs in SF and fully defined B8 and B9 medium, concerning proliferation of bovine, porcine and chicken satellite cells [98]. B8 supplemented with MC resulted in a significant GF stabilizing effect, extending the activity of GFs up to 10 days as determined by ELISA. In the short- and long-term proliferation experiments, B8 with MC, additionally supplemented with starch from corn, supported the proliferation of BSCs similar to B9, and for PSCs, a combination of B8 with MC was shown to be more effective than B9. With the price for the two excipients only about 0.1% of recombinant HSA, they allow for a substantial cost reduction for some cell lines [98].

The cost of MC and starch from corn proposed by Schenzle et al. to substitute the albumin in B9 for achieving comparable effects is assessed at USD 0.054/EUR 0.048 per Liter (as compared to USD 24.56 or EUR 21 for HSA in B9), including production costs, and these excipients are already available on the market in big quantities, are highly stable and are routinely stored at room temperature [98].

Interestingly, these stabilizers at the same concentrations were detrimental to the proliferation of fish cell lines DLEC and MACK1, underlining the differences between terrestrial and fish cell lines [98]. Similarly, recombinant human serum albumin (rHSA) produced in *Oryza sativa* and *Pichia pastoris* impaired RTgill-W1 cell viability and proliferation, reinforcing the species-specific effects of albumin [45].

Further, the addition of stabilizers to culture media allows a reduction of GF concentrations required for prolonged cell proliferation. A dilution to 70% of B8 with the basal medium DMEM-F12 and addition of either 0.1125 g/L MC with 0.4 g/L starch from corn or 0.8 g/L HSA with 0.1125 g/L MC was shown to sustain cell proliferation and high viability in short- and long-term proliferation experiments [98]. Thus, further studies on optimizing the type and concentration of GFs as well as **effective combinations of stabilizers** could result in economically viable costs for cell culture media.

Although GFs are considered as the most dominant costs drivers [94], **rHSA or BSA** in SF-media formulations designed for bovine cells also substantially contribute to costs for media (Supplementary Table S1 and Ref. [31]). Upscaling will certainly reduce the production price of CM/CSF, but other strategies are also required to alleviate the burden of costs for media. Moreover, based on assumptions by Swartz et al., millions of kilograms of albumin would be needed to produce CM that accounts for 1% of the global meat market. This quantity is by far exceeding the current production volumes of many recombinant industrial enzymes as well as serum albumins—both derived from serum (and less sustainable) and recombinantly produced [260].

#### 4.4.6. Substitution of HSA with Plant and Microbial Isolates

Substituting albumin by plant hydrolysates was already suggested by Siemensma et al. to lower shear stress for cells and to increase their robustness [191]. Results of some recent studies suggest that the replacement of albumins by plant or microbial protein extracts has the potential to be transferable to large-scale production of CM/CSF.

Non-hydrolyzed oilseed protein isolates (particularly rapeseed protein isolate, RPI) were shown to be effective albumin alternatives in SF medium Beefy-9 [261]. The isolation of RPI from rapeseed protein meal is simple and gives an ethically, environmentally, and economically sustainable stabilizer for GFs. It was shown to even exceed recombinant albumin in promoting bovine SC proliferation [261]. Therefore, it could be a viable alternative to recombinant albumins if large-scale production and storage costs remain low. However, it is hard to assess at the moment, as the product is currently not available on the market, and according to the publication the isolates must be stored at −80 °C to retain biological activity [261]. Stout et al. estimated the costs of RPI required for one Liter of medium to USD 0.002/EUR 0.0018 (without considering the isolation costs), whereas the price for commercially available recombinant albumin required for one Liter of Beefy-9 is approximately USD 24.56 or EUR 21 [31,261].

A recent report shows a simple solution allowing to avoid the necessity of −80 °C storage of RPI. Egger et al. demonstrated that lyophilized or even spray-dried protein isolates retain their biological activity if the concentration step is omitted prior to drying [262]. They further show that Styrian pumpkin protein isolate can slightly outperform RPI in short-term proliferation experiments, which demonstrates the possibility for using various local agricultural waste streams. Finally, a four times higher yield of soluble protein was achieved only by raising the grinding efficiency [262]. These findings underscore that press cake protein isolates can become a highly economical alternative to HSA. However, non-protein-based alternatives to HSA might be even cheaper (as discussed in Section 4.4.5).

Very recently, Dolgin et al. reported the effective **substitution of HSA** in B9 medium with different **microbial lysates** [206]. In this study, 0.1 g/L lysates of the yeast *Saccharomyces cerevisiae* were able to substantially improve proliferation of mackerel cell line Mack1 [74] in 2.5% FBS medium without any adaptation period. Interestingly, an HSA-free medium with 40 mg/L lysates of Gram-negative marine bacterium *Vibrio natriegens* surpassed the HSA-containing B9 medium in long-term culture of immortalized BSCs [261] after an adaptation period of 4 weeks [206]. However, the authors did not indicate the levels of endotoxin produced by *V. natriegens,* which, if present in culture medium, may represent a safety issue, although it is possible to dramatically reduce endotoxin production in *V. natriegens* using genetic engineering [263]. However, the fact that microbial lysates can substitute HSA as stabilizing agent is very remarkable.

Additionally, the same level of short-term cell proliferation (day 3) of chicken fibroblasts in SF media was achieved with water-soluble **plant proteins** extracted from soybeans, peas, and chickpeas, with only 0.1 mg/mL of plant proteins replacing 2 mg/mL of costly bovine serum albumin (BSA) (patent WO 2021/148955A1 [264]). Hemp and wheat proteins were not able to deliver comparable results. Higher amounts of plant protein extracts lead to cytotoxicity, which was eliminated after depletion of the <10 kDa fraction from the plant proteins [264]. Still, these effects have to be confirmed in the long-term, multipassaging experiments.

### 4.5. Hydrolysates as a Part of a Non-Defined Basal Medium

**Hydrolysates** obtained from **plants and microorganisms** by enzymatic or acidic digestion were discussed as a cell culture component for several decades. As discussed above, hydrolysates from plant materials can be used as a substitution of FBS for pharmaceutically relevant cell lines, such as CHO, HEK, VERO, BHK, as well as insect cell lines. For these cell lines they can further act as albumin substitutes in media to ensure high cell resistance to shear stress and high viability in bioreactors [191]. This application did not show the same efficiency for CM/CSF cell lines, allowing only partial substitution of FBS; but, due to their rich nutritional composition, they may substitute essential basal cell culture media components in CM/CSF production [188,194,265,266].

#### 4.5.1. Plant Cell Extracts as a Basal Media Substitution

Media components derived from plants can be a sustainable alternative as plants grow photoautotrophically, which reduces the environmental footprint and resource consumption [267,268,269,270].

Plant hydrolysates may be used as a nutrient source to replace components of chemically defined basal media used for the CM/CSF production, but some points should be considered. Ideally, a plant used to produce organic media components has GRAS (Generally Recognized As Safe) status but is not a food or feed crop to avoid direct competition with human and animal nutrition. At the same time, the plant should have a high biomass productivity and nutrient content, few antinutrients (e.g., phytates and lectins; [271]) and minimal demands regarding fertilization and irrigation as well as a high adaptability to different climate zones.

Importantly, the **composition of plant biomass and corresponding extracts** can vary substantially between different plant species, tissues (e.g., leaves vs. roots) over time, and depending on the cultivation and extraction conditions, contributing to batch-to-batch variations. For example, more than 20 small molecule compounds were identified by mass spectrometric analysis in *Moringa oleifera* leaf extracts [272] as well as extracts of *Sanguisorba minor* (salad burnet) prepared from either leaves/stems or roots [273]. Among these compounds, kaempferol-O-glucuronide (a flavonol reducing lipid peroxidation in animal cells [274]) was found at >20 mg/g in extracts of leaves/stems but was not detected in roots. In contrast, (+)-catechin (a flavanol stimulating the proliferation of mammalian cell cultures [275]) was detected at constant levels of 25 mg/g in all tissues.

Furthermore, light sources show effects on plant growth and metabolite accumulation. The effects of light conditions on anthocyanins, carotenoids, and flavonol concentrations in various plant species have recently been reviewed [276]. Similarly, tobacco (*Nicotiana tabacum*) leaves contained high concentrations of rutin (a flavonol that can reduce DNA damage in mammalian cell cultures [277]) if cultivated in a greenhouse, but the compound was undetectable if plants were cultivated in a phytotron using LED light [278]. The metabolite rutin was shown to stimulate proliferation of SCs isolated from Hanwoo steers [279]. The geographic location of plants also affects the molecular composition of extracts as reported for *M. oleifera* leaves for which antioxidant capacities varied between 0.5 and 1.1 mmol Fe^2+^/g [272]. Cell culture media are frequently deficient in antioxidants, although they appear to have positive effects when added to cell cultures [280]. Thus, correcting this deficiency by supplementing media with plant-derived extracts containing antioxidants can have additional beneficial effects on cells.

The composition of the plant biomass may be tailored to the requirements of cultured meat applications through selected cultivation conditions of specific plants. The best **control of cultivation conditions** to obtain a desired reproducible composition of plant extracts can be achieved by indoor farming. One such option is vertical farming, which can reduce the footprint by a factor of >5, increase biomass output two-fold and can be installed regardless of the climate at a given geographic location [281]. The most reproducible plant (cell) biomass can be obtained from plant cell suspension cultures in bioreactors. However, the number of large-scale plant cell culture processes may be too small to yield meaningful amounts of biomass from which basal media components can be extracted. The by-far largest operation is at 75 m^3^ [282], probably with an average weekly biomass output of 15,000 kg wet mass (~1000 kg dry mass). Likewise, the economic and ecological viability of such a process should be carefully assessed, because plant cell culture-based processes will typically rely on complex carbon sources like glucose too that need to be derived from other sources. Microalgae (see Section 4.5.2) can be an alternative in this context, combining the advantages of carbon fixation and control of a reactor setting.

Further, **preparation methods** have a substantial impact on the nutrient composition of hydrolysates [232,283,284]. The buffer type and concentration, pH, conductivity, temperature and presence of detergents will affect the resulting extraction efficacy for individual plant components. Hydrolysates can be produced by enzymatic digestion and/or acid hydrolysis of protein-rich plants. Plant raw materials are usually pretreated with heat before digestion or hydrolysis by enzymes or chemicals. Plant biomass can be boiled at 100 °C or more (at higher pressure) for 1 h at low pH (~1.0) to break down proteins to peptide or even amino acid level [203]. Whereas this can increase the accessibility of nutrients and inactivate pathogenic contaminations, the harsh conditions can also destroy heat sensitive molecules like vitamins [285], and lead to deamidation of amino acid residues, resulting in the conversion of neutral residues to negatively charged residues [286]. Further, many plant proteins precipitate at a pH below 5.0 and temperatures above 60 °C [287]. Enzymatic treatment at pH 5.0 and 55 °C is milder and can achieve about 90% of hydrolysis [288], but it can take up to 48 h [289], and the degree of hydrolysis may be <40% for various proteases even at high enzyme concentrations [290]. The resulting plant hydrolysates **are complex mixtures** of oligopeptides, free amino acids, vitamins and carbohydrates, minerals, and other bioactive components [233]. Regarding the composition of plant hydrolysates, further safety issues, such as bioactivity of the resulting peptides and risks of contamination have to be considered and are discussed together with an overview of preparation methods in the following detailed reviews [194,195].

Additionally, plants contain **various metabolites** that can be difficult to identify and quantify but they may have an impact on cell proliferation of mammalian cells. These metabolites are usually pooled based on the available detection assays. For example, the concentration of antioxidants can be determined via ABTS (named after the detection reagent 2,2′-azino-bis (3-ethylbenzothiazoline-6-sulfonic acid), DPPH (named after 2,2-diphenyl-1-picrylhydrazyl), and FRAP (ferric reducing ability of plasma) assays. Thus, instead of quantifying each compound individually, the respective IC_50_ values are reported for ABTS and DPPH [272].

Some plant-derived small molecules have also shown anti-cancer activity, such as paclitaxel, which has been in use for the treatment of breast cancer since the 1990s [291]. In biomanufacturing, this activity can be a drawback because these molecules may also reduce the proliferation of non-cancer cells or immortalized cells. This has been demonstrated for HeLa and HEK 293 cells [292,293], but it could be relevant for immortalized CM/CSF cell lines [54,261].

Plant-derived compounds such as sanguinarine [294], genistein (an isoflavone angiogenesis inhibitor), lycopene (a carotenoid found in many fruits), and resveratrol (a stilbene found in grape berries and therefore also in wines) [295] can be toxic to animals with a median lethal dose (LD_50_) of these compounds ranging from ~30 to >5000 mg kg^−1^ body mass (Table 3).

They probably still pose only a small risk in bioprocesses including animal cell culture because most of these toxic compounds are present at low levels in the plant biomass and are extracted predominantly in non-aqueous buffers, which are incompatible with animal cell culture applications due to toxicity at volume fractions > 20% (v·v^−1^) relevant for extraction [307,308,309].

Interestingly, all the compounds listed here have been researched at low concentrations as nutraceuticals and are considered for anti-aging and/or anti-cancer interventions [310,311]. This might open a new exciting possibility to produce CM/CSF with anti-aging or anti-cancer properties, though caution must be taken, as at least for some of them—e.g., resveratrol—detrimental effects for certain conditions were observed [312]. An interesting approach was demonstrated recently, where naturally occurring plant flavonoids curcumin, naringenin, and quercetin were shown to reduce reliance of bovine ESCs aggregates on FGF-2 during proliferation and mesodermal differentiation [313].

Moreover, some plant-extracted components were shown to have specific positive effects on muscle cells, e.g., quercetin—a plant flavonoid widely distributed in nature—boosted myogenesis, and led to increased myotube thickness and length for several species [70]; and naringenin—flavanone present in citrus fruits—promoted myogenic differentiation of porcine satellite cells (PSCs) *in vitro* and increased the content and maturity of generated myotubes [314] (for the full list Table 4).

Some authors have raised concerns that pesticides or herbicides could be present in plant extracts used in cell culture and thus pose a potential safety risk [26]. This is a relevant aspect if plants for extract preparation are cultivated in the open field. However, to ensure consistent extract quality, it seems likely that plant material destined for fermentation or cell culture applications is produced in closed cultivation facilities discussed above [281] where pesticides and herbicides are typically not used. Still, monitoring the presence of these potentially harmful substances is relevant and other sources of environmental contamination—e.g., air, water, soil, as well as those from processing—can pose issues too, making good traceability of materials important.

Plant extracts may result in reduced nutrient uptake (e.g., of iron) compared to animal-derived meat products due to reduced levels of vitamin B12 [324]. Accordingly, it will be important to test extracts from different plant types and tissues for their performance in cultured meat applications. Therefore, to obtain plant extracts with desired components for culture media used for CM/CSF production, it is also necessary to determine adequate types of starting material and to standardize hydrolysis processes.

For aquatic species, the same picture can also be observed. For example, wheat gluten hydrolysate resulted in a slight increase in fish cell proliferation when cells were cultured in Ultra-CULTURE medium, an SF medium based on DMEM/F12 supplemented with insulin, transferrin, and a purified mix of bovine serum proteins, including albumin [50]. However, higher concentrations of wheat gluten (10 g/L) led to a reduction in cell proliferation by 40–65% and soy hydrolysates were found to inhibit proliferation. These findings are consistent with previous reports by [192], which documented reduced maximal cell densities for CHO cells when high concentrations of wheat gluten hydrolysate were incorporated into the UC medium. Elevated levels of nutrients, particularly oligopeptides, can disrupt the nutritional equilibrium of the culture medium [325]. High concentrations of hydrolysates may exceed the homeostatic capacity of cells to regulate amino acid uptake and metabolism, leading to osmotic and metabolic stress—associated with increased medium osmolality and the accumulation of nitrogenous byproducts such as ammonia—rather than enhanced proliferation.

In addition to hydrolysates obtained from cultivated plants, the utilization of **hydrolysates from plant-based agro-industrial waste**, such as soybean meal, peanut meal, sunflower bran, etc., are of **special interest** regarding sustainability and costs of media compounds [194]. Other protein-rich plant-based by-products, such as press cakes, meals, rice bran, and waste potato juice [326] should be also considered as viable sources of ingredients for culture media in the future to support the principles of a circular economy and reduce environmental impact [262,270,327,328,329].

For further research in this area, **CM/CSF-relevant cell lines** should be used to ensure usability of the results—e.g., bovine primary and non-spontaneously immortalized cell lines are now commercially available, and primary cells can be easily isolated using well established protocols—e.g., in the SOP Database of the FEASTS project on FEASTS website or [31].

#### 4.5.2. Microalgae Extracts as a Sustainable Basal Medium Alternative

Microalgae may provide a sustainable supplement source for cell culture media because they store large quantities of digestible proteins and lipids. The most abundant classes of micro-algae are the diatoms in phytoplankton, the green algae, e.g., *Chlorella vulgaris*, and the golden algae [330]. The main advantage of microalgae is their content of essential amino acids, which is much higher than those from terrestrial plant proteins (see Table 1 in [331,332]. Microalgae are also a source of carbohydrates, polyunsaturated fatty acids, sterols, minerals, antioxidant vitamins, and carotenoids [331,333].

Due to their high protein content and nutritional values, microalgae extracts were studied as an alternative to replace nutrients in basal chemically defined media or to substitute FBS in animal cell culture. The capacity of algae extracts as **replacements for DMEM** was tested by Okamoto et al. with C2C12 mouse myoblasts [201] and primary bovine myoblasts [202]. Nutrients extracted from microalgae rescued cell proliferation of C2C12 mouse myoblasts cultured in a glucose- and amino acid-free medium [201].

**In the presence of serum**, replacement of DMEM by green microalgae *Chlorella vulgaris* extracts (5% of the total culture volume) resulted in the same proliferation rates for primary bovine myoblasts as in DMEM after 6 days in cell culture. Moreover, after induction of differentiation, the myotube area was comparable to that of bovine myoblasts cultured in DMEM [202]. However, excess addition of algal extracts to the cell culture medium decreased cell viability of myoblasts, indicating that algae extract also includes factors that counter its positive effects on cell proliferation.

Algal extracts obtained by acid hydrolysis contain glutamic acid [201], whereas the basal medium DMEM/F12 contains glutamine and glutamic acid (https://www.thermofisher.com/at/en/home/life-science/cell-culture/mammalian-cell-culture/cell-culture-media/dmem-f12.html, accessed on 9 June 2026). Cell viability was lower in presence of glutamic acid compared to a glutamine containing medium. Further, the concentrations of some nutrients in algae extracts, such as tryptophan and vitamins B6 and B9, were lower than those in DMEM, which may lead to rapid depletion of these basic nutrients [202].

**Cell-cultured food also requires glucose** in culture medium as a source of energy for cells. Most of the glucose is produced from starch obtained from grains such as corn and wheat, which causes negative environmental impacts. Microalgae could supply glucose that has a much smaller environmental footprint than glucose from grains [207].

One of the main **drawbacks of using microalgae** is that its protein contents can vary greatly, from few percentages up to almost 50% or higher depending on algal species, harvesting season, and different natural environments [334]. This high variability complicates standardization processes that are essential for incorporating microalgae extracts in media formulations used in CM/CSF production. Another major disadvantage is their **low doubling rates under autotrophic conditions**, ranging from 8 h to several days [335,336]. Although, that can potentially be avoided if heterotrophic growth is used instead of autotrophic growth, which at an industrial scale could lead to same or even lower costs [337,338]. However, it has to be noted that CO_2_ fixation occurs at much lower rates during heterotrophic growth, and that the proteome and metabolome of both cultivation modes is somewhat different [339], which might require additional media adaptation efforts.

#### 4.5.3. Brewer’s Spent Yeast Potential as a Part of a Non-Defined Basal Medium

Hydrolysates obtained from agro-industrial wastes like Brewer’s spent yeast could provide sustainable sources of nutrients for the cultured food sector. Moreover, this would offer opportunities to valorize waste and reduce its negative impacts on the environment [340]. Further, the yeast *Saccharomyces cerevisiae*, also commonly known as brewer’s yeast, is regarded as Generally Recognized As Safe (GRAS) [341]. Although most residual biomass from big breweries is already subjected to a cascading use in food and feed industry, smaller-scale breweries frequently dispose their spent yeast which is significantly contributing to the organic load of wastewater [342]. Integration of such local waste into the production of highly valuable cell culture components for culture medium could solve this problem.

Brewer’s spent yeast is potentially well suited as a substitute for basal medium. It contains **45–60% protein** and 40% of the total amino acids are comprised of essential amino acids, and are rich in vitamins and minerals [341,343]. Nutrient composition, however, can vary depending on extraction processes and conditions used, and consequently have different impacts on cell proliferation *in vitro*. For example, when autolysis was carried out at 47 °C, autolysates with varying free amino acid content and peptides of different molecular weights were obtained depending on the autolysis time [344]. For a comprehensive review on ingredients and production technology we refer to a review from Tao et al. [289].

In regard to the additional potential of yeast for circular bioeconomy, it is exciting to see that the search for identifying low-cost carbon sources among agro-industrial wastes (e.g., molasses, sugarcane bagasse, high fructose syrup by-products, etc.) for recombinant protein production in yeast is progressing [345,346].

As discussed in Section 4.1.3, yeast extracts were evaluated as an FBS alternative in cell culture, but the **usability of yeast-based hydrolysates** as a full or partial basal medium alternative for CM/CSF production will depend on **additional studies** regarding optimization and standardization of extraction methods for the preparation of extracts from spent brewer’s yeast. Recently, the possibility of using a mixture of yeast and plant hydrolysates as a full substitution of a DMEM/F12 basal medium was shown, which performed comparably to the control during five passages of proliferation and differentiation of BSCs [328]. Additionally, a comparative life cycle assessment showed potentially lower climate change impacts observed for HBBM, associated with a reduced contribution from energy-intensive chemical synthesis and purification processes compared to the fully defined DMEM/F12 formulation [328]. We thus conclude that the formulation of a tailor-made brewer’s yeast-based supplement for SF basal media could provide an economical and sustainable option for cell culture media, thereby enhancing the circular economy within the cellular and traditional agricultural sectors.

### 4.6. Circular Cell Culture as a Waste Reduction Strategy

Solutions to recycle or treat the enormous volume of liquid waste, i.e., spent culture medium, which will be generated during big scale CM/CSF production must be considered as it cannot be simply discarded. This would be inherently wasteful and will lead to a tremendous environmental burden and loss of valuable resources [207]: after three days in cell culture, mouse myoblasts consume 99% of glucose but only 69% of glutamine initially present in DMEM. However, the amount of 14 other amino acids decreased by only 26% [347]. In addition, more than 80% of the phosphorus in the medium was not consumed by the myoblasts after three days. The amount other medium ingredients like pyruvate or vitamins as well as minerals barely changed [347] which mainly indicates that the concentration of these components could be optimized to reduce costs. Recycling these non-consumed nutrients from spent media would allow a resource-saving cultured meat production.

Bioprocess technology approaches can be also used to recycle spent media, and applicable strategies in this direction were reviewed by Quek et al. [88]. In the following sections, the recycling approaches developed by Shimizu et al., Haraguchi et al., and Yang et al. [207,208,209] will be discussed. The potential of yeast species in medium recycling will also be discussed. This strategy would significantly reduce the requirements for regular media exchanges and the production of waste media, drastically reducing the overall medium costs.

#### 4.6.1. Microalgae and Circular Cell Culture for Sustainable Meat Production

Haraguchi et al. proposed concepts for the recycling of substances in **spent culture media** of animal cells by a **co-culture system of algae and animal cells** [347]. In this system, algae supply oxygen to animal cells, while animal cells produce carbon dioxide and ammonia, components that are nutrients for algae [347,348]. However, in this setting, animal muscle cells excreted much more lactate than ammonia, which is not a metabolite for microalgae. Therefore, a different approach was required for removing lactate from spent media. The L-lactate dehydrogenase gene from *E. coli* was introduced into **cyanobacterium** *Synechococcus* sp. PCC 7002. This genetically modified **microalgae were able to consume lactate** and convert it into pyruvate, which can be converted into glucose by mammalian cells [349]. Thus, an advanced circular cell culture (CCC) system was developed in which genetically modified *Synechococcus* microalgae and animal cells mutually exchanged substances of their metabolic waste products: (i) microalgae utilized lactate and ammonia excreted by animal muscle cells, (ii) the animal cells metabolized pyruvate and some amino acids excreted by microalgae, and (iii) rat liver cells produced GFs required to promote animal cell proliferation.

Applying this CCC system allowed mouse muscle C2C12 cells to grow efficiently for several cycles **without the need for animal serum, and without producing waste medium to be disposed** [208]. The use of microalgae is a key feature of this CCC system, which makes it possible to repeatedly use a cell culture medium, opening possibilities for a low-cost and low-environmental-load cultured food production [208]. CCC systems would allow to reduce the environmental impact of large-scale CM/CSF production and support the idea that cultured food provides a cleaner alternative to conventional meat. However, prior to upscaling, the solution for the sourcing of the GFs has to be found, the systems’ efficiency has to be demonstrated using a CM-relevant cell line instead of C2C12 over multiple recycling cycles, the quality of the final product produced in such system should be compared to the conventional meat product, and an environmental impact assessment of such process has to be performed to identify possible bottlenecks. For the latter, one project is already currently running at the University of Helsinki in collaboration with the Institute of Advanced Biomedical Engineering and Science, Japan [350]. It should also be kept in mind that the utilization of genetically modified organisms in the food industry could raise regulatory concerns, especially in the European Union.

From a scalability standpoint, several key components of the CCC system are already built upon industrially mature technologies, including utilization of *P. pastoris* for recombinant protein production [351], industrial hydrolysates generation from different source biomass [344,352], and cell culture filtration, necessary for spatial separation of both organisms and for eventual partial medium exchange [209]. The primary uncertainty pertains to the implementation of multi-cycle media recycling, which will necessitate comprehensive empirical assessment to gain a clearer understanding of the influence of factors not covered so far, including osmolarity drift, cell debris, salts accumulation, etc.

#### 4.6.2. Brewer’s Spent Yeast Potential for Circular Cell Culture

As discussed in Section 4.5.3, **yeast cell hydrolysates** could contribute constituents to **basal cell culture media**, thereby reducing the overall medium costs, and can be easily genetically modified to allow the production of GFs independent of human-consumable carbon sources. Moreover, some yeast species—e.g., brewer’s yeast—can naturally utilize such non-sugar carbons as **lactate** (unlike microalgae) [353] and use **ammonia** as a nitrogen source, e.g., to produce glutamate and glutamine [354]. Aside from that, easy handling protocols and a wide range of methods and manipulation tools are available for several yeast species [355,356]. This fulfils the essential requirements for the development of **yeast-based CCC systems**, similar to those utilizing microalgae and rat liver cells, as discussed in the previous section. First data showing experimental validation of its single components was recently demonstrated [357]. Such systems would offer the **significant advantage** of a markedly higher proliferation rate of yeasts (doubling time 1.5–2 h [358]) as compared to microalgae (doubling time 8 h to several days [335,336]). If used without co-culturing, this could offer substantial benefits in terms of space and time required for the recycler microorganism to metabolise the waste medium.

Nevertheless, regardless of circulation systems used, it should be kept in mind that the starting volumes of the corresponding media will be required. It may also be challenging to find a common culture medium for the recycler microorganisms and various cell lines relevant for CM/CSF production.

## 5. Regulatory Submissions and Industry Outlook

The CM/CSF industry has seen an increase in regulatory approvals worldwide, with Australian start-up Vow announcing its entry in Hong Kong’s and Singapore’s markets with a cultured duck foie gras [359], which followed earlier regulatory approvals in Singapore for GOOD Meat’s chicken products, and in the United States where Upside Foods and GOOD Meat have received Food and Drugs Administration (FDA) no-questions memoranda for their cultured avian meat products [360,361].

From a regulatory standpoint, culture medium formulations must meet various legal requirements regarding their chemical composition. These primarily focus on identifying hazardous compounds, compounds that pose risks of allergenicity, or compounds that are present in the final products due to their presence in media and are in concentrations not traditionally found in conventional meat products [360,361,362].

The recent regulatory engagement report from Australian start-up Vow indicates that the company has conducted extensive safety testing on specific media components, evaluating their presence in the final cell-based quail products. While exact tests were not highlighted in the public version of the report, it is indicated that there were no toxicological concerns associated with cell culture medium components [362].

In the United States, toxicological assessments for both Upside Foods and GOOD Meat included testing for heavy metals (e.g., lead, arsenic, cadmium, and mercury) and comprehensive microbiological analysis covering aerobic plate counts, coliforms, *E. coli*, *Enterobacteriaceae*, molds, *Salmonella*, *Enterobacter cloacae*, and influenza types A and B, with additional screening for a broad panel of human and bovine viruses in GOOD Meat’s submission. These results were compared to conventional ground chicken products [363,364]. Interestingly, one analysis found that cultured chicken contained higher levels of arsenic, cadmium, and lead compared to conventional chicken. Notably, the lead content was found to be 20 times higher in avian cells cultured in serum-free media than in conventional chicken tissue, underpinning the importance of incorporating the stepwise safety evaluations into upscaling processes [363].

In most regulatory submissions, companies are addressing allergenicity by assessing the presence of allergens in cultured meat products [362,364]. For instance, Vow’s culture medium, which contains barley proteins, prompted the company to test the gluten content in their cell-based quail products. Notably, the results indicated that gluten levels in the final product were below detectable limits [362]. However, available documents from regulatory bodies do not describe the concentration of gluten of the samples nor the detection limit of the method used for its quantification.

Another important consideration for culture media is its nutritional composition and the need for comprehensive safety and toxicological assessments of nutrients and compounds present in CM products, particularly when their levels differ from those found in conventional meat products. These concerns are emphasized in the FSANZ assessment of Vow’s quail products, as well as in regulatory submissions by start-ups in the U.S. [360,361,362].

Regulatory dossiers often contain full nutritional analysis of CM products that include protein, fat, ash, moisture content, as well as content of minerals, vitamins, and cholesterol, and their potential differences to conventional chicken products [363,364]. In instances where a deviation in concentration of any given nutrient is noted, a comprehensive description of its historical consumption in food by humans and potential toxicology is followed. For example, folic acid is not traditionally found in chicken products in relevant concentrations as a nutrient, and therefore safety concerns related to its content in Upside Foods’ and GOOD Meat’s cultured chicken products were addressed thoroughly in both companies’ regulatory submissions [360,361]. This was attributed to folic acid use in culture medium to support metabolic processes related with cell proliferation, while consequently accumulating in the food but nonetheless being within the concentration range of other commonly consumed foods.

A recent overview of CM-relevant GFs present in food products, such as meat and milk, was published by Huang et al. This study indicates that historically significant amounts of GFs are being consumed by humans [238]. Reported concentrations in pasteurized cow milk reached as high as 155 ng/mL EGF, which is 15 times higher than the 10 ng/L utilized in differentiation medium [128]. Concentrations ranging from 5 to 100 ng/mL found in these products are comparable to quantities in most reviewed media formulations. However, a limitation of such comparisons is the lack of published concentrations of GFs in final raw and cooked CM products, which will need to be assessed by the manufacturer during dossier preparation. For example, in the FDA’s analysis of Upside Foods’ dossier, GF safety and their sources were given attention and a request for clarification regarding their use as process aids, history of safe use, and dietary intakes. In addition, Upside Foods has analyzed GF concentration in cooked food products and noted that their concentration was below the limit of detection, highlighting that cooking process has likely denatured/destroyed the GFs used for cell culture [360,363], which is in line with the effect of heat treatment in human and cow milk on GFs [238]. Still, care must be taken, as some GFs—e.g., TGF-β, IGF-1, EGF—when stabilized by milk albumins for example, are able to withstand thermal treatments at 85 °C to a substantial degree [238]. Ultimately, each process should be tailored according to the choice of growth factor and bioprocess parameters optimized for optimal growth or protein content, while ensuring product safety.

In addition to folic acid, GOOD Meat’s FDA dossier highlighted medium components that could be present in their products though not explicitly covered by regulation for use as process additives. The list included ferric nitrate, hypoxanthine, thymidine, lipoic acid, putrescine, sodium pyruvate, polyoxamer 188, and FBS [364]. The company has shown the prevalence of most of these components in common foods at similar or lower concentrations. In the case of the non-ionic surfactant polyoxamer 188, it is stated that its content in final products (due to carryover from media) was within the dosage reported in the literature where no adverse effects have been witnessed [361,364], and in one cultured chicken product it was as low as 1/75th of the acceptable daily intake [364].

In the case of CSF, similar approaches towards ensuring safety of medium for food applications must be carried by the companies. Interestingly, the first regulatory submission documents from the company Wildtype, concerning the safety assessment of the medium used to grow their salmon cells, highlights a process akin to that of CM companies [365]. Firstly, the company highlights components that are not present as ingredients in the food supply, calculate the expected concentration in their products and daily intake analysis, and finally compare them to established thresholds based on scientific literature. The compounds assessed in the study included freezing agents, nicotinamide adenine, and taurine [365].

Findings from regulatory submissions underscore region-agnostic visions around food safety regarding culture media components in CM. Recently, a comprehensive report has identified trends in regulatory assessments in different regions, providing a summary of public guidelines available from multiple regulatory agencies globally [366]. So far, Gourmey and Mosa Meat have submitted applications to European Union regulators for authorization of their respective products, but no regulatory dossiers are available yet, thereby hindering a more in-depth analysis of the regulatory process in the region [13,367].

## 6. Key Performance Indicators

Key performance indicators (KPIs) are specific metrics that highlight the most essential areas of an organization’s performance—those that directly influence its present effectiveness and long-term success [368]. When applied to nascent industries, KPIs provide insights into technological maturity, such as product development or regulatory milestones, and guidance for establishing benchmarks to track a company’s progress toward market readiness.

Based on current knowledge from literature cited and discussed throughout this manuscript, expert discussions and KPI validations as well as recent analysis and reports [17,54,359,363,366,369], the following culture media-related KPIs for current CM/CSF processes have been defined:

- High media to biomass conversion ratios: as CM processes develop towards commercial feasibility, it is important that most media inputs added should generate as much biomass as possible in order to optimize process cost. Therefore, the most biomass generated from the least medium volume or weight possible is often ideal and can be used as a KPI to track medium performance. These conversion ratios can be calculated from the kg or L of media consumed to produce a given amount of CM, such as 1 kg, according to the following equation: (Medium-to-biomass ration) = (Total biomass produced)/(Medium consumed). Values for media-to-biomass conversion ratios has been described in the literature for an animal-free medium used to grow chicken embryonic fibroblasts, highlighting that roughly 65 L of media were required to produce 1 kg of CM. Similar values to this can be used as benchmarks for this KPI, while expecting further optimization to improve profitability of CM processes and achieve price parity.
- Medium cost: one of CM/CSF highest costs is culture medium and its contribution to CM/CSF final cost should be in the order of cents of EUR per L to reach cost parity with conventional meat [17]. However, it is dependent on the process and the conventional counterpart it attempts to emulate. A more accurate unit of measurement for this KPI at scale is media cost per mass of CM/CSF (EUR/kg). This measurement can be calculated from the cost of medium per L multiplied by the kg of CM produced. For the commercial feasibility of non-premium CM (excluding high-cost delicacies like foie gras and bluefin tuna) it is expected that medium cost should be under 30% of total cost, and is expected to be the highest contributor to the cost-of-goods, with a study mentioning as much as 60% [17]. While there is a lack of empirical studies highlighting medium costs in commercial settings, some authors have described values that allow commercial feasibility of CM. For instance, a custom-made serum-free medium for embryonic chicken fibroblasts that allows their continuous growth in high cell densities represented a cost of USD 0.63 per L, or USD 40 per kg of wet cell biomass [17], which in turn allows production of hybrid CM below the cost of conventional organic chicken.
- Number of population doublings supported by proliferation media in relatively low doubling times (<30 h): adequate medium performance is needed (upwards of 60 population doublings) to sustain a process from cell bank to large bioreactor expansion. The number of population doublings can be calculated according to the following equation: PD = (log(N_t_)-log(N_0_))/log(2), where N_t_ the final number of cells after expansion phase and N_0_ the initial number of cells. Some processes described in the literature highlight media capable of withstanding continuous growth of CM cells for more than 100 population doublings [17].
- Animal component-free and food-safe formulation: free of animal components like FBS, bovine serum albumin (BSA), as well as from GFs that are not specific to the animal product intended to produce. Formulation should take regulatory constraints in entry market(s) into account.
- Waste medium recycling/waste management concept.
- Antibiotic-free formulation: Media formulations free of antibiotics and antimycotics, and residual presence (under exposure limits for adverse effects to arise) or complete absence from final products is expected.
- Hazard Analysis and Critical Control Points (HACCPs) in place, quality control and quality assurance (QA/QC), and sterility plans for bulk media production. Media components and formulations should be assessed on their contamination risks when not fully chemically defined, and batch-to-batch variability thresholds should be put in place for ill-defined components and mixtures. Furthermore, growth factor stability in different culture settings should be assessed, according to production process.
- Supply chain plans: supply-chain redundancy and resiliency for medium component sourcing.

## 7. Conclusions

Enormous quantities of cell culture media will be required for large-scale production of CM and CSF. Therefore, a central economic question concerns the costs of medium as it significantly contributes to the price of CM and CSF products, which should reach price parity with conventional meat and seafood in the not-too-distant future.

Cell culture media need to be high-performing in terms of cell proliferation and differentiation and should be free of any animal-derived material due to ethical and safety concerns (see Table 5). In a recent survey conducted for this review, six out of seven companies offering media for CM and CSF development or production confirmed that animal-derived ingredients are not applied. These media are generally made up of a basal medium and several supplements including GFs. However, final medium composition depends very much on the type of cell lines and species. According to the survey (see Supplementary File S2), companies prefer to customize media for their specific cell lines over available standard media.

However, medium optimization still faces challenges as there is lack of data regarding essential nutrients and GFs for single cell lines from various animal species (see Table 5). Once essential components are identified for specific cell lines, their concentration must be optimized. The amount of GFs—one of the main cost drivers for media—could be reduced by prolonging their short half-life by increasing proteolytic stability through mutations, microencapsulation or with stabilizing compounds. In this respect, costly albumins could be substituted with low-cost methylcellulose or protein isolates as stabilizing agents [98]. Based on our survey, the majority (60%) of companies supplement media with naturally occurring (non-engineered) GFs, which may be linked to possible regulatory issues arising from application of engineered GFs. Some authors suggest that an “in-house” production of stabilized and highly bioactive cell-type specific GFs may facilitate cost savings as well [196]. The identification of effective minimal GF variants as shown for HGF by deNola [221] could further reduce manufacturing costs for CM and CSF, though regulatory aspects of using mutant variants are not clear (see Table 5).

A critical element for an economically viable CM and CSF production is the substitution of costly cell culture medium reagents with low-cost and food grade alternatives. In this regard, hydrolysates from plants and microorganisms represent nutrient rich sources of cell culture medium ingredients (see Table 5). According to the survey, two out of seven companies offer CM/CSF media containing lysates or hydrolysates derived from plants or microalgae. Even waste or by-products of agro- and food industries could provide proteins, peptides, vitamins or carbohydrates required for basal media. To avoid high batch-to-batch variations of compounds extracted from alternative sources, extraction/preparation methods need to be standardized through the establishment of Standard Operating Procedures.

Further, new technologies for medium recovery or recycling will be essential to prevent the waste medium generated during CM/CSF production processes from having a negative environmental impact (see Table 5). In this regard, circular cell culture (CCC) systems are promising. In a novel CCC system developed by Haraguchi et al., microalgae supply nutrients for mammalian cells and at the same time serve as a waste-medium recycler [370]. Mixed cell cultures have long been used for industrial applications [371]. Improving culturing success by using a multi-species co-cultured or parallel cultured system hold enormous potential, but this approach is complex and requires extensive optimization.

Current approaches regarding SF media often depend on empirical methods and utilize formulations originally designed for non-food areas or model cell lines used in research, which may not be well-suited for the specific requirements of cell lines proposed for the CM/CSF production. Optimizing one factor at a time is often used to design new media formulations, but this strategy is very inefficient considering the high number of media components, e.g., the basal medium DMEM/F12 consists of 52 components [27]. Further, possible interactions among media components remain unexplored with this approach [372]. The throughput capacity of medium optimization studies can be enhanced by using statistical experimental designs and the application of high-throughput systems [373]. Design-of-Experiments (DoE) methods can manage large numbers of components in fewer experiments and allow the evaluation of a wider design space to identify optimal conditions [372,373]. In general, DoE methods can optimize < 10 variables [374], and when including screening designs > 25 variables could be considered [375]. The DoE approach together with a Response Surface Methodology approach was successfully applied by Skrivergaard et al. when developing an SF medium for the proliferation of bovine muscle cells [30], and by Schenzle et al. for optimization stabilizer combinations for GFs [98].

To deal with increasing complexity, the use of artificial intelligence (AI)/machine learning (ML) for media optimization could be promising for developing single cell line specific culture conditions for CM/CSF production (see Table 5). ML approaches are generally used to develop predictive models based on the relationships among features of a given dataset. ML commonly involves processing input data, training of an appropriate model, and predicting new data to be evaluated in lab experiments [265,376]. Recently, a strategy for SF medium optimization for CM was proposed, incorporating multi-omics profiling, systems biology, multi-scale metabolic modeling, ML, and real-time monitoring, adaptive feeding-guided SFM formulation [377]. ML-based approaches have been already successfully applied for medium development for mammalian cell cultures [378,379,380]. An AI approach was also applied to optimize medium formulations for fish cells [381]. The resulting formulation with optimized quantities of GFs, selenium, and ascorbic acid, and a reduced amount of FBS could enhance zebrafish cell proliferation rate by >50%, suggesting that AI has the potential to accelerate the development of animal-free medium formulations.

Further proposed applications of AI and ML for CM research include microscopy and image analysis for medium and cell line development, as well as bioprocessing optimization—areas that are well established within medical research and biotechnology [382]. Additionally, ML in thermal reaction modeling for flavor design has been recently proposed for cultured fat applications [383].

Beyond that, ML-based protein structure prediction tools, such as AlphaFold, ProteinMPNN, or Rosetta [384,385,386], may in the future be employed to accelerate the identification of animal-free or low-priced alternatives to GFs, protein-based stabilizers (HSA/BSA/fetuin), or adhesion proteins (e.g., see Section 4.4.4). Such tools are already being used for engineering therapeutic proteins [385,387], predicting more stable or biologically active variants by modeling protein–protein and protein–ligand interactions necessary, e.g., for modeling receptor activation. These are highly potent tools; however, experimental validation of the results is essential. Caution should also be exercised when selecting the validation method, as a greater affinity to the receptor does not necessarily equate to increased biological activity [388]. However, applying these AI/ML technologies to develop highly efficient cell culture media requires a deeper understanding of the physiological and nutritional needs of mammalian and fish cell lines under *ex vivo* conditions and the collection of high throughput data from transcriptomic, proteomic, and metabolic analyses [378].

As media composition depends on multiple factors like cell type, species, density, and types of essential supplements, and as companies producing CM/CSF customize and hide their medium formulations, a quantification of expected cost reductions is difficult. Companies offering media for the CM/CSF area stated that prices range from ≤1 up to EUR 100/L. According to our survey, five out of seven companies can offer media for CM production with ≤1 up to EUR 5/L, whereas only two out of seven companies offer media for CSF in this range. It is still about a 100-fold difference as compared to the cents of EUR per L, which are considered necessary to reach cost parity with conventional meat, as we state in Section 6. Taking strategies proposed for cell culture media cost reductions mentioned in this review into account, an economically viable and sustainable production of CM/CSF should be possible in the near future.

## Figures and Tables

**Table 1:** Media used for cell isolation after biopsy.

Species	Basal Medium	Supplements	References
Bovine SCs	DMEM	20% FBS + 1% penicillin-streptomycin	[65]
Bovine SCs	DMEM	10% FBS	[66]
Bovine SCs	DMEM	10% FBS + 1% antibiotic and antimycotic mix	[67]
		2% L-glutamine	
Bovine SCs	DMEM + Glutamax	20% FBS + 1% primocin	[31,68]
		human FGF-2 (1 ng/mL)	
Bovine SCs	DMEM	10% FBS + 1 × antibiotic-antimycotic	[69]
Bovine, porcine, and chicken SCs	Ham’s F-10	20% FBS + 1% penicillin and streptomycin	[70]
		mouse FGF-2 (5 ng/mL)	
Porcine SCs	DMEM	10% FBS + penicillin (100 U/mL) + streptomycin (100 µg/mL) + gentamicin (20 µg/mL)	[71]
		2% porcine serum	
Porcine SCs	Alpha MEM Eagle	20% FBS + 1% penicillin–streptomycin + 1% amphotericin B + 0.5% gentamicin	[72]
Bovine adipocytes	DMEM	10% FBS + 1% penicillin/streptomycin/amphotericin	[73]
Fish SCs	L-15	20% FBS + 1% antibiotic–antimycotic + 10 µg/mL gentamicin	[74]
		1 ng/mL human FGF	
		20 mM HEPES	
Fish myoblasts	L-15	20% FBS + 100 µg/mL penicillin/streptomycin	[75]
		10 ng/mL IGF	
		10 ng/mL EGF	
		10 ng/mL bFGF	
		0.8 mM CaCl_2_	
		2 mM glutamine	
Fish adipocytes	DMEM/L-15 (1:1)	10% FBS + 1% antibiotic/antimycotic solution	[76]

**Table 3:** Examples of toxic compounds identified in plant extracts. Plant species potentially useful for cell culture media component extraction based on their food status are highlighted in **bold font**.

Plant Species	Compound	Concentration in Biomass [mg/kg Dry Mass]	Reference	LD50 [mg/kg]	Organism	Route of Administration	Reference
*Sanguinaria canadensis*	Sanguinarine	1400	[296]	1658	Rat	Oral	[297]
*Sanguinaria canadensis*	Sanguinarine	1400	[296]	29	Rat	Intravenous	[297]
*Berberies vulgaris*	Berberine sulfate	20,000	[298]	205	Rat	Intraperitoneal	[299]
*Berberies vulgaris*	*Berberies vulgaris* root extract	20,000	[298]	1280	Rat	Oral	[299]
***Glycine max***	Genistein	4.6	[300]	>2000	Rat	Oral	[301]
***Glycine max***	Genistein	4.6	[300]	300	Mice fibroblasts Balb/c 3T3 cell line	*In vitro*	[302]
***Solanum lycopersicum***	Lycopene	300	[303]	>5000	Rat	Oral	[303]
***Vitis vinifera***	Resveratrol	50–700	[304]	5	Human LX-2 hepatic stellate cells	*In vitro*	[305]
***Vitis vinifera***	Resveratrol	50–700	[304]	>3000	Rat	Gavage	[306]

**Table 4:** Substances/compounds promoting cell proliferation/differentiation.

Compounds (Natural Source)	Mechanism of Action	Effect on Cells	Cell Type Tested	References
**Quercetin** (vegetables, fruits, herbs)	Myostatin inhibitor.	Best results with 10 and 100 nM quercetin; promotion of proliferation; conflicting reports on **differentiation**; increased myotube thickness or length.	Bovine, pig, chicken SCs, bovine ESCs, and C2C12	[70,313]
**Licochalcone A and B** (*Glycyrrhiza uralensis*)	Myostatin inhibitor.	Enhance muscle **proliferation** and **differentiation.**	Bovine, porcine, chicken MSCs, C2C12	[70]
**Gingerol, Curcumin** (ginger, curcuma)	Attenuate the effect of advanced glycation end products on myoblasts.	Both promote **differentiation** at concentrations of 1 μM and 5 μM; curcumin supports both proliferation and differentiation of ESCs.	C2C12, bovine ESCs	[313,315]
**Rosmarinic acid** (aromatic plants)	Activates the unfolded protein response and the mTOR pathway.	Enhances CHO cell **proliferation** and viability (final conc. 0.5 mM).	CHO	[316]
**Rutin** (various fruits, plants)	Stimulate Pax7 and/or MyoD.	Stimulation of cell **proliferation** (100–200 μM); no effect on differentiation.	Bovine SCs from Hanwoo steers	[279]
**Parthenolide** (plant feverfew)		Stimulation of cell **proliferation** (0.5–1 μM); no effect on differentiation.		
**Naringenin** (tomato, grapefruit, and citrus fruits)	Upregulates the IGF-1/AKT/mTOR anabolic pathway during myogenesis of SCs by activating the estrogen receptor β.	Naringenin (20 μM) promotes myotube formation (**differentiation**) and maturation in porcine SCs, and early expansion of bovine ESCs.	Porcine SCs, bovine ESCs	[313,314]
**Vitamin K2** (vegetables, fish, meat, cheese, egg)	Accelerates transfer of electrons in mitochondria, leading to more efficient ATP production and reduced LDH release into the media.	Vitamin K2 (10 μM) improves **proliferation** and migration of bovine skeletal muscle cells.	Bovine SCs	[317]
**Vitamin D** (fish and mammals)	Promotes the expression of myogenic markers and GFs, myotube formation; upregulates the expression of follistatin (myostatin inhibitor), and downregulates expression of myostatin.	Vitamin D (100 nM 1,25-D3) exerts a pro-myogenic effect on satellite cells (promotion of **differentiation**).	SCs from mice	[318]
**β-carotene** (fruits and vegetables)	Increase PPARγ mRNA expression.	Enhancement of adipogenesis (**differentiation**).	Bovine white adipocytes	[172]
**Lutein** (fruits and vegetables)				
**Spermidine** (present in all plant and animal derived food types)	Linked to biological processes which modulate cellular proliferation, differentiation and apoptosis.	Enhancement of proliferation rate.	Bovine SCs	Patent WO 2021/158103 [319]; [320]
**Sericin** (silk protein; waste in silk industry)	Mitogenic factor involved in cell proliferation and attachment.	Accelerates the proliferation, replacement of FBS in SF-media.	Bovine SCs	Patent US 2022/0098546A1 [321]; [322,323]

**Table 5:** Key challenges that remain to be addressed (white spaces).

Topic	To Do
Food grade and animal component-free media, especially for proliferation of aquatic cell lines, and isolation/cryopreservation of primary cells	Depending on species (e.g., bovine, porcine, avian, fish) and cell types, define **essential medium components** for isolation, proliferation, and differentiation.
Food safe proliferation-promoting components	Depending on species (e.g., bovine, porcine, avian, and especially fish) and cell types, identify **appropriate type of GF** or proliferation-promoting compound.
	Engineer **minimal active and stable GF** versions.
	Identify low-cost **stabilizers for GFs or immobilization strategies.**
	Define **regulatory and safety constrains** for media components (species-specific/not? Wild-type/mutants? Naturally occurring/artificial? Safe rest concentrations? Reliable methods for quantification?)
Low-cost carbohydrate sources	**Analyze** the potential of agro-waste products (e.g., molasses).
Low-cost protein sources (peptide or amino acid)	Analyze the potential of agro-waste products (e.g., brewer’s yeast, plant hydrolysates).
Low-cost-free fatty acids	Analyse the potential of plant-based oils including agro-waste
Development of SOPs	**Standard** extraction methods for different nutrients (e.g., proteins, vitamins, lipids) from natural sources or waste.
Waste medium recycling/waste management	Optimize **recycling of non-consumed** media components (glucose/amino acids/vitamins and minerals, albumins, growth factors).
	Develop techniques to **remove/upcycle harmful cellular metabolites,** e.g., ammonia and lactate.
CCC-systems using mixed cell cultures	Establish and optimize a fully animal-source independent, highly efficient **multi species cell consortia** for CCC systems applicable for CM/CSF production processes.
AI/ML-based optimization of cell culture medium	Collect data and establish stable **models for AI/ML** approaches.

## Data Availability

Data are contained within the article and Supplementary Materials.

## Supplementary Materials

The following supporting information can be downloaded at: https://www.mdpi.com/article/10.3390/foods15142494/s1, File S1: Supplementary Tables. Supplementary Table S1: SF proliferation media formulations, costs, components, and their concentrations; Supplementary Table S2: SF myogenic media formulations, costs, components, and their concentrations; Supplementary Table S3: SF adipogenic media formulations, costs, components, and their concentrations; anonymous survey on media for cultured meat/seafood production. File S2: Anonymous Survey on media for cultivated meat/seafood production.

## Abbreviations

The following abbreviations are used in this manuscript:

AA	Arachidonic acid
ABTS	2,2′-azino-bis(3-ethylbenzothiazoline-6-sulfonic acid)
APE	*Auxenochlorella pyrenoidosa* extract
B8	Basal 8 medium
B9	Beefy-9 medium
BHK	Baby hamster kidney cells
BMP4	Bone morphogenetic protein 4
BSA	Bovine serum albumin
BSCs	Bovine satellite cells
CCC	Circular cell culture
CHO	Chinese hamster ovary cells
CM/CSF	Cultured meat/cultured seafood
C2C12	Mouse myoblast cell line
cAMP	Cyclic adenosine monophosphate
cGMP	Cyclic guanosine monophosphate
DHA	Docosahexaenoic acid
DEX	Dexamethasone
DMEM/F12	Dulbecco’s Modified Eagle Medium/Ham’s F-12
DMSO	Dimethyl sulfoxide
DPPH	2,2-diphenyl-1-picrylhydrazyl
E8	Essential 8 medium
EGF	Epidermal growth factor
EPA	Eicosapentaenoic acid
ERK1/2	Extracellular signal-regulated kinases 1/2
ESCs	Embryonic stem cells
F10	Ham’s F-10 medium
FAPs	Fibro-adipogenic progenitor cells
FABP11	Fatty acid-binding protein 11
FATP1	Fatty acid transport protein 1
FBS	Fetal bovine serum
FGF-1/2	Fibroblast growth factor 1/2
GF/GFs	Growth factor(s)
GRAS	Generally Recognized As Safe
GSK3β	Glycogen synthase kinase 3 beta
HEPES	4-(2-hydroxyethyl)-1-piperazineethanesulfonic acid
HEK	Human embryonic kidney cells
HGF	Hepatocyte growth factor
HSA	Human serum albumin
IBMX	1-methyl-3-isobutylxanthine
IC50	Half maximal inhibitory concentration
IGF-1/2	Insulin-like growth factor 1/2
IGF-1R	IGF-1 receptor
IL-6	Interleukin-6
ITSE	Insulin–transferrin–selenium–ethanolamine
L-15	Leibovitz’s L-15 medium
LIF	Leukemia inhibitory factor
LPL	Lipoprotein lipase
LPAR1	Lysophosphatidic acid receptor 1
MC	Methylcellulose
MSCs	Mesenchymal stem cells
NO	Nitric oxide
NRG-1	Neuregulin 1
PD	Population doublings
PDGF-BB	Platelet-derived growth factor BB
PLO	Poly-L-ornithine
PLGA	Polylactic glycolic acid
PPARγ	Peroxisome proliferator-activated receptor gamma
PSCs	Porcine satellite cells
PUFA	Polyunsaturated fatty acids
rHSA	Recombinant human serum albumin
SCs	Satellite cells
SF	Serum-free
T3	Triiodothyronine
TGF-β1/TGF-β3	Transforming growth factor beta 1/3
TFRC	Transferrin receptor
TNFα	Tumor necrosis factor alpha
VERO	Vero cell line (African green monkey kidney cells)
VEGF	Vascular endothelial growth factor
WNT	Wingless/Integrated signaling pathway
