# Supplementary material for: Towards Cost-Effective and Sustainable Media Formulations for Terrestrial and Aquatic Cellular Agriculture
Source: Foods. 2026 Jul 14;15(14):2494. doi: 10.3390/foods15142494 (PMC13407990; doi:10.3390/foods15142494)
Supplement: Supplementary file 1 [file foods-15-02494-s001.zip › foods-4366982-supplementary.pdf]

Supplementary materials, Supplementary file S1:

Review

Towards Cost-Effective and Sustainable Media Formulations For Terrestrial and Aquatic Cellular Agriculture

Regina Leber [1], Joana T. Rosa [2,3], Vincent Laizé [2,3], Gonçalo F. Fernando [4], Johannes Buyel [5] and Aleksandra Fuchs [1,6,*]

1 ACIB—Austrian Centre of Industrial Biotechnology, Petersgasse 14, 8010 Graz, Austria;
2 S2AQUA—Collaborative Laboratory, Association for a Smart and Sustainable Aquaculture, Av. Parque Natural da Ria Formosa s/n, 8700-194 Olhão, Portugal; joana.rosa@s2aquacolab.pt (J.T.R.)
3 CCMAR— Algarve Centre of Marine Sciences, University of Algarve, Campus de Gambelas, 8005-139 Faro, Portugal; vlaize@ualg.pt (V.L.)
4 Associate Laboratory i4HB—Institute for Health and Bioeconomy, Instituto Superior Técnico, Universidade de Lisboa, Av. Rovisco Pais, 1049-001 Lisbon, Portugal; goncalo.fernando@tecnico.ulisboa.pt
5 Institute of Bioprocess Science and Engineering (IBSE), Department of Biotechnology and Food Sciences (DBL), BOKU University, Muthgasse 18, 1190 Vienna, Austria; johannes.buyel@boku.ac.at
6 Division of Physiology, Otto Loewi Research Center for Vascular Biology, Immunology and Inflammation (E.O.), Medical University of Graz, 8010 Graz, Austria
* Correspondence: al.fuchs@medunigraz.at; Tel.: +43-316-385-73856

**Medium**	**E8**	**B8 and B8 + MC + starch from corn**	**B9**	**Kolkmann**	**Skrivergaard**	**Pasitka SF 2022 medium**	**ACF medium&**	**Component (bulk) cost**	**Product information**
Cell type (Ref.)	Human stem cells [1]	Human stem cells [2], BSCs, PSCs [3]	BSCs [4]	Bovine SCs [5]	Bovine SCs [6]	Chicken fibroblasts [7]	Chicken fibroblasts [8]		
DMEM/F12 *	1	1	1	1	1	1	1	€24/L	Thermo Fisher #11320033
ITSE Animal-Free**	-	-	-	1%	-	-	-	€488.64/100 mL	InVitria 777ITS032
Not animal-origin free ITS**	-	-	-	-	1%	-	-	€55.5/10 mL	Thermo Fisher #41400045
rInsulin	19.4 mg/L	20 mg/L	20 mg/L	**<u>10 mg/L</u>**	**<u>10 mg/L</u>**	3 mg/L	-	€41,920/100 g	Upon request from InVitria
Ascorbic acid 2-phosphate	64 mg/L	200 mg/L	200 mg/L	50 mg/L	-	-	-	€513/100 g	Sigma #49752
rTransferrin	10.7 mg/L	20 mg/L	20 mg/L	**<u>5.5 mg/L</u>**	**<u>5.5 mg/L</u>**	-	-	€70/g	Upon request from Oryzogen
Sodium selenite	14 µg/L	20 µg/L	20 µg/L	**<u>6.7 µg/L</u>**	**<u>6.7 µg/L</u>**	7 µg/L	7 µg/L	€240/100 g	Sigma # S5261
rFGF-2 (FGF-2-G3***)	100 µg/L	40 µg/L***	40 µg/L	10 µg/L	2 µg/L	10 µg/L	-	€22/mg	Upon request from Oryzogen
rTGFß1	2 µg/L	0.1 µg/L	0.1 µg/L	-	-	-	-	€1,235/100 µg	Upon request from Oryzogen
rNGR1	-	0.1 µg/L	0.1 µg/L	-	-	-	-	€1,484.00/mg	Upon request from Peprotech
rVEGF	-	-	-	10 µg/L	-	-	-	€150/mg	Upon request from Oryzogen
rIGF-1	-	-	-	100 µg/L	-	-	-	€30/mg	Upon request from Oryzogen
rHGF	-	-	-	5 µg/L	20 µg/L	-	-	€4,735.00/mg	Thermo Fisher #100-39-01M
rPDGF-BB	-	-	-	10 µg/L	5 µg/L	-	-	€3,845.00/mg	Thermo Fisher #100-14B-1MG
rhIL-6	-	-	-	20 µg/L	-	-	-	€2,965/mg	Thermo Fisher #200-06-1MG
Sodium bicarbonate	543 mg/L	2428 mg/L	2428 mg/L	-	-	-	-	€59.30 per 500 g	Sigma #S5761
HSA	-	-	800 mg/L	5 g/L	-	-	-	€25/g	Upon request from Oryzogen
BSA	-	-	-	-	75 mg/L	-	-	€154/100 mL 7.5% in DPBS (x100)	Sigma #A8412
rFibronectin	-	-	-	10 mg/L	-	-	-	€35/mg	Upon request from Oryzogen
Hydrocortisone	-	-	-	36 µg/L	-	2 mg/L	-	€305/10g	Sigma # H0888
L-alanine-L-glutamine	-	-	-	-	-	2 mM	4 mM	€18.700,71/50 L of 200 mM	Sartorius SKU: 03-022-1B, upon request
Glucose	-	-	-	-	-	-	5 g/L	€170,10/25 kg	Laboratoriumdiscounter #GLUC1.3
Methylcellulose (MC)	-	0.01%§	-	-	-	-	0.1%	€339/kg	Sigma #M0512
Others		• 0.4 g/L starch from corn§		• 1 mg/L α-linoleic acid<br>• **<u>0.2 g/L ethanolamine</u>**<br>•1 % GlutaMAX	• 600 mg/L fetuin	• 10 mg/L in-house lipid mixture§	• 10 mg/L in-house lipid mixture§<br>• 7 U/ml antioxidant solution&&<br>• 0.3 mg/ml HPBCD§§§	• Starch from corn €221.00/5 kg<br>• Alpha-linoleic acid €1,680/10 g<br>• GlutaMAX €77/100 mL<br>• Fetuin €3,240/25 g<br>• HPBCD €1,470/100 g<br>• § Price?	• Starch from corn Sigma #S4126<br>• Alpha-linoleic acid Sigma #L2376<br>• Fetuin Sigma #F2379-25G<br>• GlutaMAX Thermo Fisher #35050061<br>• HPBCD§§§§ Sigma #H107-100G
**Price per Liter (no basal medium)**	**€36.17**	**€12.70<br>€12.75§**	**€32.70**	**€658.18<br>€308.18§§**	**€262.67<br>€148.74§§§**	**€5.30**	**€12.29**		

Supplementary Table S1*:* SF proliferation media formulations, costs, components and their concentrations. Basal medium costs – DMEM-F12 – were not considered. Bulk prices – when available – are included.

* DMEM/F12 (Thermo Fisher #11320) contains L-glutamic acid (7.35 mg/L) and L-glutamine (365 mg/L).

** 100x ITS formulations contain 1 g/L insulin, 0.55 g/L transferrin and 0.67 mg/L sodium selenite, and ITSE additionally contains 0.2 g/L ethanolamine. If ITS/ITSE is included into formulation, components concentrations contained therein are marked **<u>bold underlined</u>**, and only ITS/ITSE costs are considered.

*** In commercially available B8, provided by Defined Biosciences, a stabilized version of FGF-2 [9] is used.

§ B8 additionally stabilized by methylcellulose (MC) and starch from corn. §§ Without fibronectin. §§§ Without HGF and PDGF-BB. §§§§ HPBCD - 2-hydroxypropyl-β-cyclodextrin.

& ACF medium seem to contain undefined GF(s), listed in Fig 2b [8], which we include into this calculation with the listed price of $0.008/L = €0.0071/L.

&& Undefined composition, undefined price.

	Myogenesis				
**Component**	**McAleer**	**Messmer**	**MyoFusion**	**Component (bulk) cost**	**Product information**
Cell type (Ref.)	Rat myoblasts [10]	BSCs [11]	BSCs [12]		
Basal medium, to 1L	Neurobasal : L15= 1:1	DMEM/F-12	DMEM		
rEGF	100 µg/L	10 ng/ml	-	€371.41/mg	Fisher #50-197-6254
rIGF-1	10 µg/L	-	-	€225.00/mg	Qkine #Qk047
TGF-β1	-	-	4.5 ng/ml	€5,915/mg	Cell Guidance Systems #GFH39-1000
PDGF-BB	-	-	4.66 ng/ml	€2,965/mg	Thermo Fisher #200-06-1MG
rHuman serum albumin	-	0.5 mg/ml	-	€25/g	Upon request from Oryzogen
L-ascorbic acid 2-phosphate	-	40 µM	-	€513/100 g	Sigma #49752
MEM AA solution	-	1x	-	€546.00/2000 ml of 50x	Fisher #11580386
$NaHCO_3$		6.5 mM	-	€59.30 per 500 g	Sigma #S5761
Sodium selenite	-	80 nM	-	€240/100 g	Upon request from Oryzogen
rInsulin	-	1.8 µM	-	€41,920/100 g	Upon request from InVitria
Lysophosphatidic acid (LPA)	-	1 µM	-	€212.57/25 mg	Focus #10-1449
Cytosine arabinoside	-	-	7.3 ng/ml	€96.55/g	Adooq #A10265
Linoleic acid	-	-	0.207 µg/ml	€639/kg	Sigma W338020-1KG
rTransferrin	-	135 nM	-	€70/g	Upon request from Oryzogen
Acetylcholine	-	10 µM	-	€313.00/500 g	TCI #A0084
**Price per Liter (no basal medium)**	**€39.39**	**€26.95**	**€40.40**		

Supplementary Table S2: SF myogenic media formulations, costs, components and their concentrations. Basal medium costs were not considered. Bulk prices – when available – are included.

Adipogenesis								
Porcine and ovine FAPs [13]			Chicken fibroblasts [7]			Stromal-vascular cells of red sea bream [14]		
**Components**	**Component (bulk) cost**	**Product information**	**Components**	**Component (bulk) cost**	**Product information**	**Components**	**Component (bulk) cost**	**Product information**
DMEM/F-12	1 L		DMEM10	1 L		DMEM/F12 (1:1)	1 L	
HEPES 4.9 mM	€3,290/5 kg	Sigma #H3375-5KG	200 µM oleic acid	€1,082.90/100 g	Thermo #270291000	0.042 mg/L linoleic acid	€639/kg	Sigma W338020-1KG
Lipid concentrate 0.1%	€100/100 ml	Thermo #11905031	12 µg/ml L-α-phosphatidylcholine (soy lecithin)	€820.00/g	Sigma #P7443-1G	65 mM NaCl	€162.75/25 kg	Roth #3957.5
Putrescine 57 µM	€120.95/50 g	Roth #4141.3				50 µg/mL rTransferrin	€70/g	Upon request from Oryzogen
Progesterone 17.8 nM	€105.70/25 g	Glentham #GP3663-25g				5 ng/mL sodium selenite	€240/100 g	Upon request from Oryzogen
Hydrocortisone 25 nM	€305/10 g	Sigma #H0888				50 ng/mL hydrocortisone	€305/10 g	Sigma # H0888
Calcium Chloride 1 mM	€162.50/25 kg	Roth #A119.5				50 µg/ml bovine insulin	€3,162/g	Cell Appl.#128-1000
L-Ascorbic acid 2-phosphate 227 µM	€513/100 g	Sigma #49752						
Glucose 17 mM	€170.10/25 kg	Laboratoriumdiscounter #GLUC1.3						
FGF-2 2 ng/ml	€22/mg	Upon request from Oryzogen						
EGF-1 2 ng/ml	€371.41/mg	Fisher #50-197-6254						
BMP-4 10 ng/ml	€4,140/mg	Thermo #120-05ET-01M						
rInsulin 10 µg/ml	€41,920/100 g	Upon request from InVitria						
Rosiglitazone 5 µM	€284.85/200 mg	Fisher # 16481246						
**Price per Liter (no basal medium)**	**€60.53**		**Price per Liter (no basal medium)**	**€10.45**		**Price per Liter (no basal medium)**	**€161.63**	

Supplementary Table S3: SF adipogenic media formulations, costs, components and their concentrations. Basal medium costs were not considered. Bulk prices – when available – are included.

References:

1. Chen G, Gulbranson DR, Hou Z, et al. Chemically defined conditions for human iPSC derivation and culture. *Nat Methods*. 2011;8(5):424-429. doi:10.1038/nmeth.1593

2. Kuo HH, Gao X, DeKeyser JM, et al. Negligible-Cost and Weekend-Free Chemically Defined Human iPSC Culture. *Stem Cell Rep*. 2020;14(2):256-270. doi:10.1016/j.stemcr.2019.12.007

3. Schenzle L, Egger K, Spangl B, et al. Low-cost food-grade alternatives for serum albumins in FBS-free cell culture media. *Sci Rep*. 2025;15(1):15296. doi:10.1038/s41598-025-99603-7

4. Stout AJ, Mirliani AB, Rittenberg ML, et al. Simple and effective serum-free medium for sustained expansion of bovine satellite cells for cell cultured meat. *Commun Biol*. 2022;5(1):466. doi:10.1038/s42003-022-03423-8

5. Kolkmann AM, Van Essen A, Post MJ, Moutsatsou P. Development of a Chemically Defined Medium for in vitro Expansion of Primary Bovine Satellite Cells. *Front Bioeng Biotechnol*. 2022;10:895289. doi:10.3389/fbioe.2022.895289

6. Skrivergaard S, Young JF, Sahebekhtiari N, et al. A simple and robust serum-free media for the proliferation of muscle cells. *Food Res Int*. 2023;172:113194. doi:10.1016/j.foodres.2023.113194

7. Pasitka L, Cohen M, Ehrlich A, et al. Spontaneous immortalization of chicken fibroblasts generates stable, high-yield cell lines for serum-free production of cultured meat. *Nat Food*. 2022;4(1):35-50. doi:10.1038/s43016-022-00658-w

8. Pasitka L, Wissotsky G, Ayyash M, et al. Empirical economic analysis shows cost-effective continuous manufacturing of cultivated chicken using animal-free medium. *Nat Food*. 2024;5(8):693-702. doi:10.1038/s43016-024-01022-w

9. Dvorak P, Bednar D, Vanacek P, et al. Computer-assisted engineering of hyperstable fibroblast growth factor 2. *Biotechnol Bioeng*. 2018;115(4):850-862. doi:10.1002/bit.26531

10. McAleer CW, Rumsey JW, Stancescu M, Hickman JJ. Functional myotube formation from adult rat satellite cells in a defined serum-free system. *Biotechnol Prog*. 2015;31(4):997-1003. doi:10.1002/btpr.2063

11. Messmer T, Klevernic I, Furquim C, et al. A serum-free media formulation for cultured meat production supports bovine satellite cell differentiation in the absence of serum starvation. *Nat Food*. 2022;3(1):74-85. doi:10.1038/s43016-021-00419-1

12. Tavsanli A, Milkevych V, Young JF, Rasmussen MK. Designing the MyoFusion Media: A Serum-Free Medium Optimized for Bovine Satellite Cell Differentiation. *Biotechnol Bioeng*. Published online May 13, 2026. doi:10.1002/bit.70237

13. Mitić R, Cantoni F, Börlin CS, Post MJ, Jackisch L. A simplified and defined serum-free medium for cultivating fat across species. *iScience*. 2023;26(1):105822. doi:10.1016/j.isci.2022.105822

14. Oku H, Tokuda M, Okumura T, Umino T. Effects of insulin, triiodothyronine and fat soluble vitamins on adipocyte differentiation and LPL gene expression in the stromal-vascular cells of red sea bream, Pagrus major. *Comp Biochem Physiol B Biochem Mol Biol*. 2006;144(3):326-333. doi:10.1016/j.cbpb.2006.03.008

Supplementary File S2

Anonymous Survey on media for cultivated meat/sea-food production

7 responses

For which species do you provide cultivation media?

7 out of 7 answered

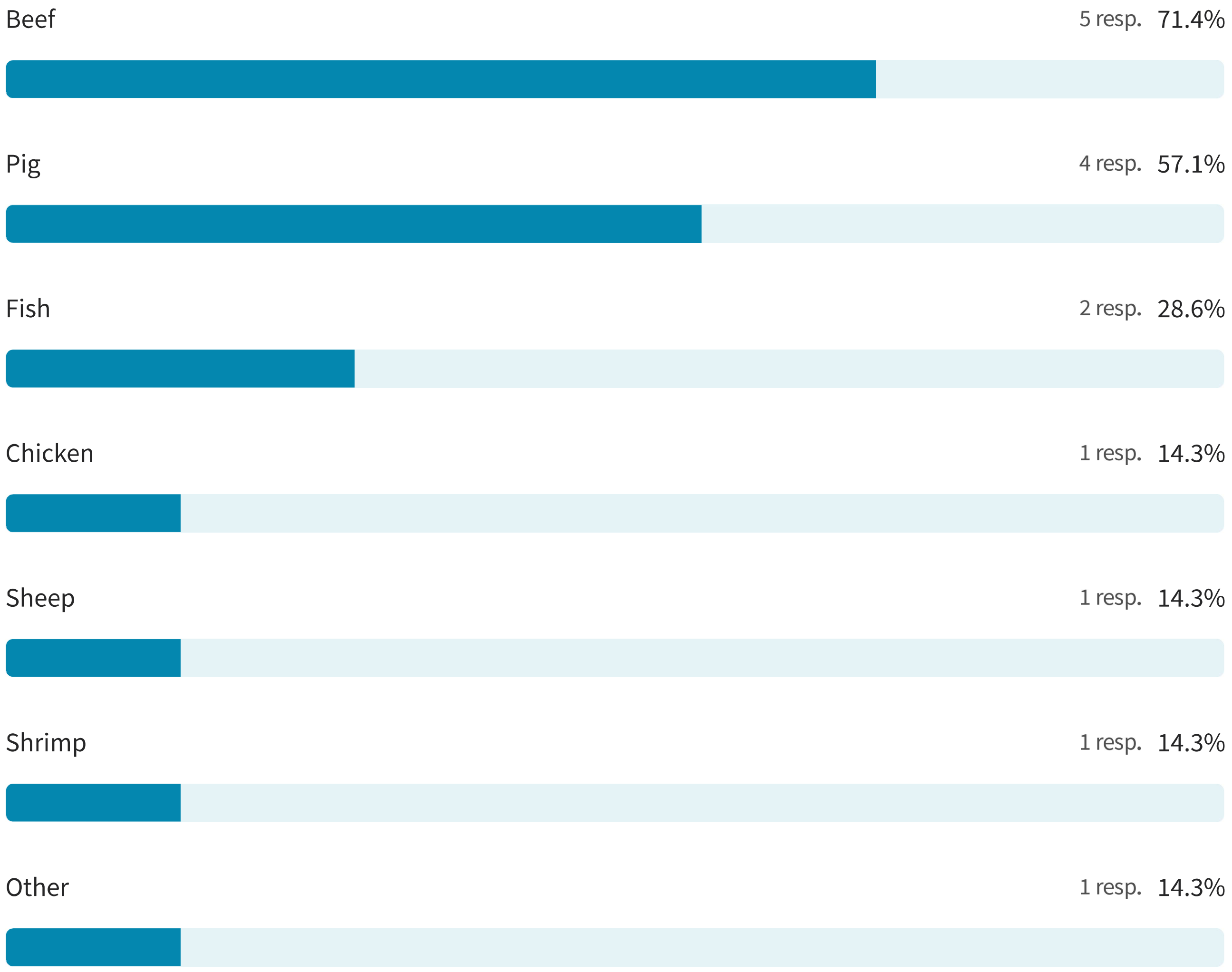

Human

If other, which?

2 out of 7 answered

Human

We offer a wide range of mineral-salts, which can be used as nutrient or technological aid for every biotech application.

What is your typical pricing for proliferation media optimized for cultivated meat production (Euro per Liter)?

7 out of 7 answered

Option	Responses	Percent
1-5 Euro/L	3 resp.	42.9%
20-50 Euro/L	2 resp.	28.6%
5-10 Euro/L	2 resp.	28.6%
≤1 Euro/L	2 resp.	28.6%
10-20 Euro/L	1 resp.	14.3%

50-100 Euro/L 1 resp. 14.3%

What is your typical pricing for proliferation media optimized for cultivated fish/seafood production (Euro per Liter)?

3 out of 7 answered

Option	Responses	Percentage
1-5 Euro/L	1 resp.	33.3%
20-50 Euro/L	1 resp.	33.3%
5-10 Euro/L	1 resp.	33.3%
50-100 Euro/L	1 resp.	33.3%
≤1 Euro/L	1 resp.	33.3%
10-20 Euro/L	0 resp.	0%

Do companies producing cultivated meat/fish additionally order optimized cultivated meat/fish media for other stages of production other than proliferation?

5 out of 7 answered

Yes 4 resp. 80%

No 1 resp. 20%

If yes, for which?

5 out of 7 answered

Differentiation 3 resp. 60%

Cryopreservation 1 resp. 20%

Isolation 1 resp. 20%

Maturation 1 resp. 20%

No, only for proliferation 1 resp. 20%

Do companies producing cultivated meat/fish prefer your standard cultivated meat/fish media, or do they prefer to customize it for their specific cell lines?

6 out of 7 answered

they prefer to customize 6 resp. 100%

they prefer your standard media 0 resp. 0%

Do companies focus more on a universal cultivated meat/fish medium for different species, or do they purchase / customize media for various species?

6 out of 7 answered

different media for various species 3 resp. 50%

universal medium 3 resp. 50%

Does your cultivated meat/fish media contain animal-derived components?

7 out of 7 answered

no 6 resp. 85.7%

yes 1 resp. 14.3%

If yes, which?

2 out of 7 answered

FBS 1 resp. 50%

Serum Albumin 1 resp. 50%

Transferrin 1 resp. 50%

Fetuin 0 resp. 0%

Other 1 resp. 50%

None

Do you use DMSO in your cryopreservation media or a food grade alternative?

6 out of 7 answered

DMSO 4 resp. 66.7%

food grade alternative 2 resp. 33.3%

Other 0 resp. 0%

If other, which?

0 out of 7 answered

Nobody answered this question yet

Regarding growth factors: Do you incorporate stable mutants of growth factors or wild type proteins to supplement your cultivated meat/fish media?

7 out of 7 answered

yes, wild type growth factors 4 resp. 57.1%

yes, stable mutants 3 resp. 42.9%

no incorporation of growth factors 2 resp. 28.6%

Do you incorporate human or mouse growth factors into your terrestrial cell lines cultivated meat media?

7 out of 7 answered

no 5 resp. 71.4%

yes 2 resp. 28.6%

What is the percentage of human or mouse growth factors of all growth factors you use for terrestrial cell lines media?

7 out of 7 answered

0% 5 resp. 71.4%

10%-40% 2 resp. 28.6%

100% 0 resp. 0%

40%-60% 0 resp. 0%

60%-80% 0 resp. 0%

80-99% 0 resp. 0%

Do you incorporate human or mouse growth factors into your aquatic cell lines media?

4 out of 7 answered

no 4 resp. 100%

yes 0 resp. 0%

What is the percentage of human or mouse growth factors of all growth factors you use for terrestrial cell lines media?

7 out of 7 answered

0% 5 resp. 71.4%

10%-40% 2 resp. 28.6%

100% 0 resp. 0%

40%-60% 0 resp. 0%

60%-80% 0 resp. 0%

80-99% 0 resp. 0%

Do you produce growth factors in house or buy from suppliers?

6 out of 7 answered

buy from suppliers 4 resp. 66.7%

both 2 resp. 33.3%

in house 0 resp. 0%

Are the applied growth factors purified?

6 out of 7 answered

partly 4 resp. 66.7%

yes 2 resp. 33.3%

no 0 resp. 0%

In which grade do you offer most of your cultivated meat/fish media components?

7 out of 7 answered

food grade 3 resp. 42.9%

pharma grade 2 resp. 28.6%

chemical grade 1 resp. 14.3%

other: 1 resp. 14.3%

feed grade 0 resp. 0%

If pharma present among answers, what is the percentage of pharma-grade media components?

3 out of 7 answered

10%-40% 2 resp. 66.7%

40%-60% 1 resp. 33.3%

0% 0 resp. 0%

100% 0 resp. 0%

60%-80% 0 resp. 0%

80-99% 0 resp. 0%

Do you offer cultivated meat/fish media containing lysates of plant/algae/yeast or other lysates or hydrolysates?

7 out of 7 answered

no 5 resp. 71.4%

yes 2 resp. 28.6%

If yes:

2 out of 7 answered

microalgae 1 resp. 50%

plant 1 resp. 50%

animal byproducts 0 resp. 0%

other: 0 resp. 0%

yeast 0 resp. 0%

If other, which?

0 out of 7 answered

Nobody answered this question yet

Do you incorporate antibiotics/antifungals into your cultivated meat/fish media?

7 out of 7 answered

no 6 resp. 85.7%

yes 1 resp. 14.3%

Do you incorporate steroid hormones into your cultivated meat/fish media?

7 out of 7 answered

no 6 resp. 85.7%

yes 1 resp. 14.3%

Do you incorporate insulin into your cultivated meat/fish media?

7 out of 7 answered

yes 5 resp. 71.4%

no 2 resp. 28.6%

Powered by Typeform
